# Geometrical inequalities bounding angular momentum and charges in General Relativity

**DOI:** 10.1007/s41114-018-0014-7

**Published:** 2018-07-05

**Authors:** Sergio Dain, María Eugenia Gabach-Clement

**Affiliations:** Facultad de Matemática, Astronomía y Física, Universidad Nacional de Córdoba, Instituto de Física Enrique Gaviola, Consejo Nacional de Investigaciones Científicas y Técnicas, Córdoba, Argentina

**Keywords:** Geometrical inequalities, Black holes, Ordinary objects

## Abstract

Geometrical inequalities show how certain parameters of a physical system set restrictions on other parameters. For instance, a black hole of given mass can not rotate too fast, or an ordinary object of given size can not have too much electric charge. In this article, we are interested in bounds on the angular momentum and electromagnetic charges, in terms of total mass and size. We are mainly concerned with inequalities for black holes and ordinary objects. The former are the most studied systems in this context in General Relativity, and where most results have been found. Ordinary objects, on the other hand, present numerous challenges and many basic questions concerning geometrical estimates for them are still unanswered. We present the many results in these areas. We make emphasis in identifying the mathematical conditions that lead to such estimates, both for black holes and ordinary objects.

## Introduction

Geometrical inequalities in General Relativity, that is, relations play an important role in understanding some physical systems. The basic questions behind these inequalities are the following. What are the reasons that such inequalities do exist at all? Another, more humble but more practical and hopefully illuminating question is about the elements in Einstein theory, that produce such inequalities. In other words: Why should we expect such inequalities, and where do they come from? The straightforward answer is that there are a number of different objects predicted by the theory that live in different regimes.

This is clear when one thinks about one of the most important class of solutions to Einstein equations: Black holes. In the evolution of stars, there are two natural limits to consider. One of them is the maximum mass a star can have, beyond which it collapses into a black hole. This problem was addressed in the 1930s by Chandrasekhar (see Wali 1982). The other is the maximum charge and/or angular momentum a black hole can have, beyond which it becomes a naked singularity. This problem arose after Reissner (1916) and Nordström (1918) found the solution describing a static, spherically symmetric, electrically charged object.

These thresholds in physical parameters values can be identified as limit cases of appropriate geometrical inequalities. In more general terms, we can naively imagine a function *f* depending on the physical parameters of the system, like the mass *M*, size *R*, angular momentum *J*, electromagnetic charge *Q*, etc., denoted by $$f:=f(M,R,J,Q, \ldots )$$, such that when *f* takes values in $$[f_{obj}^-,f_{obj}^+]$$, the system describes a non-black-hole ordinary material object, like a star or soccer ball. When *f* takes values in $$ [f_{bh}^-,f_{bh}^+]$$, it describes a black hole, and when *f* takes values in $$ [f_{nak}^-,f_{nak}^+]$$, a naked singularity.

We emphasize that this is a very rough and overly simplified picture of what the geometrical inequalities found so far actually say, and of what to expect for more general systems. But, as we see in Sects. 4.2 and 5.2, the above division showing the different regimes in which the system can exist, is what one actually finds in some cases.

In this article, we address systems containing ordinary material objects and/or black holes. The latter, and the frontier between black holes and naked singularities, is the original and main motivation for the geometrical inequalities presented here. Therefore, we explore it in what follows in the case of a paradigmatic black-hole solution.

Consider the Kerr–Newman black hole with positive mass *M*, angular momentum *J* and electric charge *Q* (see Wald 1984). The area *A* of the horizon is given by

1 $$\begin{aligned} A=4\pi \left( 2M^2-Q^2+2M \sqrt{d} \right) , \quad d= M^2-Q^2-\frac{J^2}{M^2}. \end{aligned}$$

The equality (1) implies the following three important inequalities among the parameters:

2 $$\begin{aligned}&\sqrt{\frac{A}{16\pi }}\le M, \end{aligned}$$

3 $$\begin{aligned}&\frac{Q^2+\sqrt{Q^4+4J^2}}{2} \le M^2, \end{aligned}$$

4 $$\begin{aligned}&4\pi \sqrt{Q^4+4J^2} \le A. \end{aligned}$$

These inequalities saturate in the two relevant limit values for the parameters: the Schwarzschild black hole given by $Q=J=0$ [where (2) reaches the equality] and the extreme Kerr–Newman black hole given by $d=0$ (where the inequalities (3) and (4) reach the equality). Note that inequality (3) is equivalent, by a simple computation, to the condition $d\ge 0$.

It is important to recall that the Kerr–Newman metric is a solution of Einstein electrovacuum equations for any choice of the parameters (*M*, *J*, *Q*). However, it represents a black hole (and hence the area *A* of the horizon is well defined) if and only the inequality (3) holds. Otherwise the spacetime contains a naked singularity.

Above, we have derived inequalities (2)–(4) from a very particular exact solution of Einstein equations: the Kerr–Newman stationary black hole. However, remarkably, these inequalities remain valid (under appropriate assumptions) for fully dynamical black holes. Moreover, they are deeply connected with the expected properties of the global evolution of Einstein equations, in particular with the cosmic censorship conjecture.

The inequalities (2)–(4) can be divided into two groups:

1. $\sqrt{\frac{A}{16\pi }}\le M$: the area appears as lower bound.
2. $\frac{Q^2+\sqrt{Q^4+4J^2}}{2} \le M^2$ and $4\pi \sqrt{Q^4+4J^2} \le A$: the angular momentum and the charge appear as lower bounds.

This division seems rather unnatural at first, due to the quantities involved being the same and the fact that inequalities (2) (in the first group) and (4) (in the second group) look like intermediate inequalities of (3) (in the second group). However, at the moment the division makes sense because the mathematical methods used to study these two groups are in general different. We expect that in the future new connections will appear between all these inequalities.

As the title of this article suggests, we will focus on the second group. For dynamical black holes, the inequality (2) in the first group is the Penrose inequality. There exists already an excellent and up to date review on this subject by Mars (2009).

Furthermore, the inequalities can also be classified as global or quasilocal. We explain this distinction in more detail in Sect. 2. Roughly speaking, the total ADM mass *M* is a global quantity, in contrast the area *A* is quasilocal, it depends on a bounded region of the spacetime. In contrast, there are both global and quasilocal definitions of charge *Q* and angular momentum *J*. With this in mind, (2) and (3) are global inequalities, and (4) is a purely quasilocal inequality. Global inequalities can be interpreted as refinements of the positive mass theorem in the presence of a black hole.

Our main interest in this article is to examine inequalities (2)–(4) for dynamical black holes and also in related or more general situations like stationary black holes with surrounding matter fields, ordinary objects, higher dimensions and alternative theories of gravity. However, in this article there are some topics that are left uncovered. Some of them are:

- *Geometrical inequalities involving quasilocal mass* This is a very broad subject as there are many different notions of quasilocal mass and energy. The problem of determining a unique appropriate notion that will give general useful and representative geometrical inequalities is open. There is a beautiful review by Szabados (2004), on quasilocal quantities, and in particular, quasilocal mass, that discusses this issue
- *Geometrical inequalities for black holes in higher dimensions and within alternative theories of gravity* This topic has been growing during the last years and many very interesting results have been obtained. See the work of Gibbons and Holzegel (2006), Gibbons (1999), Hollands (2012), Yazadjiev (2013a, b), Fajman and Simon (2014), Alaee et al. (2016, 2017a, b), Alaee and Kunduri (2014, 2016), Rogatko (2014, 2017), and references therein.

The results presented in this article can be grouped essentially in three parts. The first two parts concern black holes. Global inequalities of the form (3) are reviewed in Sect. 3, and quasilocal inequalities of the form (4) are presented in Sect. 4. In Sect. 4.3 we discuss a partial relation between the global and the quasilocal problems. The third part, in Sect. 5 addresses geometrical inequalities for non-black-hole objects. The mathematical methods used to study the various problems are similar in many ways but the physical implications and scopes of these types of inequalities appear to be very different, we will address this issue in the following sections.

There are also a number of articles reviewing the subject of geometrical inequalities that include some of the results presented here. They were written by Dain (2011, 2012, 2014a), and Jaramillo (2013) with slightly different approaches and focuses.

### Motivation from stationary black holes

Before discussing the general setting, it is important to analyze, in a heuristic way, the physical meaning of the inequalities (2)–(4) for stationary black holes.

Let us begin with inequality (2). This inequality describes the most basic property of a black hole, that is, its mass is concentrated in a small region of space. More precisely, in terms of the areal radius $R:=\sqrt{A/4\pi }$, this inequality is expressed as

5 $$\begin{aligned} \frac{ R}{2}\le M. \end{aligned}$$

Inequality (5) can be interpreted as a weak version of the Hoop conjecture (Thorne 1972), in which the area is taken as a measure of the size of the black hole. See Senovilla (2008) for alternative formulations and references to previous works on the conjecture, and the more recent articles by Malec and Xie (2015), Yoshino (2008), Gibbons (2009), Khuri (2009), Murchadha et al. (2010), Hod (2015) and Cvetic et al. (2011b). Interestingly, there is a related conjectured inequality introduced by Yodzis et al. (1973) and known as the trapped surface conjecture. It states that is the mass *M* enclosed in a region of size *R* does not satisfy $M\le R$ then the region must be trapped.

Consider the second inequality, (3). Using a mixture of classical and relativistic equations, in the following we will argue that this inequality is essentially a consequence of (5). Take a sphere of radius *R* with constant electric density and total charge *Q*. The classical electromagnetic energy of this sphere is given by

6 $$\begin{aligned} W_Q =\frac{3}{5}\frac{Q^2}{R}. \end{aligned}$$

In addition, suppose that the sphere has mass *M*, constant density and it rotates with constant angular velocity. The Newtonian kinetic energy of the sphere is given by

7 $$\begin{aligned} W_J = \frac{5}{4}\frac{J^2}{MR^2}, \end{aligned}$$

where *J* is the angular momentum of the sphere.

Assume that the sphere collapses and forms a black hole. Since the resulting black hole is supposed to be stationary, we can identify the ADM mass with the mass of the resulting black hole (Ashtekar et al. 2000a) and have that it should be greater than the sum of the energies

8 $$\begin{aligned} W_Q+W_J \le M. \end{aligned}$$

We use the inequality (5) to bound the radius *R* by the mass in the energies $W_Q$ and $W_J$, we obtain

9 $$\begin{aligned} \frac{3}{10}\frac{Q^2}{M} + \frac{5}{16}\frac{J^2}{M^3} \le \frac{3}{5}\frac{Q^2}{R} + \frac{5}{4}\frac{J^2}{MR^2}= W_Q+W_J. \end{aligned}$$

Hence using (8) we finally get

10 $$\begin{aligned} \frac{Q^2}{M}+\frac{J^2}{M^3} \lesssim M. \end{aligned}$$

Note that inequality (10) is equivalent to the condition $d\ge 0$ given in (1) and hence we recover the inequality (3). The symbol $\lesssim$ in (10) means that in the left-hand side of this equation we have approximated all the numerical factors that appear in Eq. (9) by one. These numbers depend on some attributes we have chosen for the sphere: i.e., constant charge and mass density. We can not expect, by this kind of argument, to obtain the precise numerical factors involved in the inequality (3), only the order of magnitude. However, remarkably, we have obtained the correct functional dependence on the parameters.

Finally, consider the last inequality (4). In the following, using thermodynamics arguments for stationary black holes (Wald 2001), we will argue that this inequality is a consequence of the inequality (3) and the existence of extreme black holes [i.e., black holes with non-zero area that saturates (3)].

Consider a general stationary black hole which is not necessarily Kerr–Newman, for example a stationary black hole surrounded by a ring of matter. Assume that there exists a function of the form $A=A(M,J,Q)$ that relates the parameters of the black hole. We can include more parameters into this function without altering the following argument. If we identify the area *A* as the entropy of the black hole, then, in the thermodynamical language, the function *A*(*M*, *J*, *Q*) would be identified as the fundamental equation of the system. Its existence is one of the postulates of Thermodynamics (see, for example, Callen 1985). The inverse of the temperature of the system is defined as the partial derivative of *A*(*M*, *J*, *Q*) with respect to *M*, and it is a positive quantity. That is, if we define $\kappa$ by

11 $$\begin{aligned} \frac{\partial A}{\partial M} = \frac{8 \pi }{\kappa }, \end{aligned}$$

we have $\kappa \ge 0$. Hence, *A*(*M*, *J*, *Q*) is an increasing function of *M* for fixed *J* and *Q*.

Assume, in addition, that we have a lower bound for the mass *M* in terms of *J* and *Q* like the inequality (3). We do not need the particular form given by (3), we assume some general inequality of the form

12 $$\begin{aligned} M\ge M_0(J,Q), \end{aligned}$$

where $M_0(J,Q)$ is a strictly positive given function. Consider the function *A*(*M*, *J*, *Q*) for fixed *J* and *Q*. The bound (12) implies that for *M* only values with $M\ge M_0$ are allowed. Since *A* is an increasing function of *M* we obtain

13 $$\begin{aligned} A(M,J,Q)\ge A(M_0,J,Q)=A_0(J,Q), \end{aligned}$$

where we have defined the function $A_0(J,Q)$ by

14 $$\begin{aligned} A_0(J,Q)=A(M_0(J,Q),J,Q). \end{aligned}$$

In order to obtain from (13) a non-trivial inequality we need to assume that $A_0>0$ (in principle we could have $A_0$ identically zero). That is, we need to assume the existence of non-trivial extreme black holes: black holes for which the bound (12) is saturated and have non-zero area.

Given that the function *A*(*M*, *J*, *Q*) ends at the value $A_0$, one can ask what happens at that point. For the extreme black hole the temperature is zero, hence the derivative (11) is infinite and then the function *A*(*M*, *J*, *Q*) can not be extended in any smooth way beyond the point $A_0$. This can be explicitly checked in the Kerr–Newman case given by (1).

We have obtained the inequality (13), which has the same general form as (4). Clearly, in order to obtain the explicit form of (4), one must take the Kerr–Newman case in the limit values $M_0$ and $A_0$, that is

15 $$\begin{aligned} M_0^2(J,Q)=\frac{Q^2+\sqrt{Q^4+4J^2}}{2}, \quad A_{0}(J,Q)= 4\pi \sqrt{Q^4+4J^2}. \end{aligned}$$

However, inequality (13), obtained with the Assumptions (11) and (12) does exhibit some general features similar to (4), namely, it suggests that the area is bounded below by the angular momentum and charges. Also, that extremal black holes play a key role in determining the minimum value for the area. And finally that the minimum value for the mass affects the minimum value for the area.

In summary, these informal results show that inequalities (2)–(4) are partially motivated by the Kerr family of stationary black holes. But there is another interesting observation in the above arguments. Namely, that Penrose inequality (2) implies the global inequality (3) and the global inequality implies the quasilocal inequality (4)

16 $$\begin{aligned} M\ge \sqrt{\frac{A}{16\pi }}\Rightarrow \quad M^2\ge \frac{Q^2+\sqrt{Q^4+4J^2}}{2} \Rightarrow \quad A\ge 4\pi \sqrt{Q^4+4J^2} . \end{aligned}$$

We note that we use the *uncharged* and *non-rotating* version of Penrose inequality (see Mars 2009 for a review on the subject and Khuri et al. 2017 for a recent result on the charged Penrose inequality for multiple black holes). The first implication in (16) may be relevant when extending the Penrose inequality to include angular momentum and charge. As we will see, the treatments of (3) and (4) are similar in some ways and in fact, a version of the second implication is obtained in the general dynamical scenario (see Sect. 4.3).

The implications (16) also put the Penrose inequality in a very especial place as being, in a sense, more basic than the other inequalities. We come back to this issue in Sect. 1.2 where it is deduced from standard arguments in collapse scenarios, and in Sect. 1.3 where it is a result of Newtonian considerations with the only condition that the speed of any particle should be smaller than or equal to the speed of light.

### Heuristic arguments in dynamical black-hole regimes

The extension of the inequality (2) for dynamical black holes was done by Penrose (1973) using a remarkable physical argument that connects global properties of the gravitational collapse with geometric inequalities on the initial conditions. We briefly review this argument below (see also Mars 2009; Dain 2012, 2014a and references therein)

We will assume that the following statements hold in a gravitational collapse:

(i) Gravitational collapse results in a black hole (weak cosmic censorship).
(ii) The spacetime settles down to a stationary final state. Furthermore, at some finite time after the collapse, all the non electromagnetic matter fields have fallen into the black hole and hence the exterior region is electro-vacuum.

Conjectures (i) and (ii) constitute the standard picture of the gravitational collapse.

The black-hole uniqueness theorem implies that the final state postulated in (ii) is given by the Kerr–Newman black hole (we emphasize however that many important aspects of black-hole uniqueness still remain open, see Chruściel et al. (2012) for a recent review on this problem). Let us denote by $M_{{\textit{KN}}}$, $A_{{\textit{KN}}}$, $J_{{\textit{KN}}}$ and $Q_{{\textit{KN}}}$ the mass, area, angular momentum and charge of the remainder Kerr–Newman black hole. These quantities will, of course, satisfy the three inequalities (2)–(4).

Let us consider an initial data set for a gravitational collapse such that the collapse has already occurred on the data. That means that the initial spacelike surface $\varSigma$ intersects the event horizon of the black hole. The intersection is a spacelike, closed, 2-surface denoted by $\mathcal {S}$ with area $A(\mathcal {S})$. Let *M* be the total mass of the spacetime defined by (69).

From the black-hole area theorem (Hawking 1971; Chruściel et al. 2001) we have that the area of the black hole increases with time and hence

17 $$\begin{aligned} A \le A_{{\textit{KN}}}. \end{aligned}$$

Since gravitational waves carry positive energy, the total mass of the spacetime (ADM) should be bigger than the final mass of the black hole (Wald 1984)

18 $$\begin{aligned} M_{{\textit{KN}}} \le M. \end{aligned}$$

Combining (17) and (18) and the fact that the remainder black hole satisfies the inequality (2), namely

19 $$\begin{aligned} \sqrt{\frac{A_{{\textit{KN}}}}{16\pi }} \le M_{{\textit{KN}}}, \end{aligned}$$

we finally conclude that

20 $$\begin{aligned} \sqrt{\frac{A}{16\pi }}\le M. \end{aligned}$$

There is still an important issue to be discussed: how to estimate the area $A(\mathcal {S})$ in terms of geometrical quantities that can be computed from the initial conditions. Recall that in order to know the location of the event horizon the entire spacetime is needed. Assume that the surface $\varSigma$ contains a future trapped 2-surface $\mathcal {S}_0$. By a general result on black-hole spacetimes (Penrose 1965; Hawking 1971), we know that the surface $\mathcal {S}_0$ should be contained in $\mathcal {S}$. But that does not necessarily mean that the area of $\mathcal {S}_0$ is smaller than the area of $\mathcal {S}$. Consider all surfaces $\tilde{\mathcal {S}}$ enclosing $\mathcal {S}_0$. Denote by $A_{\min }(\mathcal {S}_0)$ the infimum of the areas of all such surfaces. Then we clearly have that $A(\mathcal {S})\ge A_{\min }(\mathcal {S}_0)$. The advantage of this construction is that $A_{\min }(\mathcal {S}_0)$ is a quantity that can be computed from the Cauchy surface $\varSigma$. Using this inequality we finally obtain the Penrose inequality

21 $$\begin{aligned} \sqrt{\frac{ A_{\min }(\mathcal {S}_0)}{16\pi }} \le M. \end{aligned}$$

In the time symmetric case (i.e. when $K_{ij}=0$) an important simplification occurs. For that case, a marginally trapped outermost surface is a minimal surface (see Sect. 2.2 for definitions), and hence we do not need to consider the family of enclosing surfaces. For further discussion we refer to Mars (2009) and references therein.

The key point in the previous argument is that there exist simple inequalities that relate the quantities *M* and *A* on the initial conditions with the quantities $M_{{\textit{KN}}}$ and $A_{{\textit{KN}}}$ on the remainder black hole, where the geometrical inequalities are satisfied. For the second inequality (3) we need to consider the electric charge and the angular momentum. The problem is that there is no simple inequality, like (18), that relates the total electric charge and angular momentum of the initial data $(Q_\infty ,J_\infty )$ with the corresponding quantities of the final, Kerr–Newman black hole $(Q_{{\textit{KN}}},J_{{\textit{KN}}})$. We need additional assumptions.

Suppose that in the exterior of the black hole the matter fields are not charged. That is

22 $$\begin{aligned} \nabla _\mu T^{{ EM}\, \mu \nu }=0, \end{aligned}$$

on $\varSigma {\setminus } \mathcal {S}$, where $T^{{ EM}\, \mu \nu }$ is the energy momentum tensor field of electromagnetism. Then, the electric charge is conserved in the exterior region (see Sect. 2) and hence we have

23 $$\begin{aligned} Q_\infty =Q_{{\textit{KN}}} \end{aligned}$$

Using (23) and the fact that the remainder black hole satisfies

24 $$\begin{aligned} |Q_{{\textit{KN}}}|\le M_{{\textit{KN}}}, \end{aligned}$$

we get

25 $$\begin{aligned} |Q_\infty |\le M. \end{aligned}$$

That is, we have obtained the dynamical version of the inequality (3) for the case $J=0$. Note that we have not used the area theorem. We see in Sect. 3 that the assumption that the matter fields are non-charged in the black-hole exterior region can be slightly relaxed: it can be assumed that the electric charge density is small with respect to the mass density.

To relate the initial angular momentum $J_\infty$ with the final angular momentum $J_{{\textit{KN}}}$ is much more complicated. Angular momentum is in general non-conserved. There exists no general simple relation between the total angular momentum $J_\infty$ of the initial conditions and the angular momentum $J_{{\textit{KN}}}$ of the final black hole. For example, a system can have $J_\infty =0$ initially, but collapse to a black hole with final angular momentum $J_{{\textit{KN}}}\ne 0$. We can imagine that on the initial surface there are two matter regions with opposite angular momentum, one of them falls into the black hole and the other escapes to infinity. Axially symmetric vacuum spacetimes constitute a remarkable exception because the angular momentum is conserved in electrovacuum. That is, we have

26 $$\begin{aligned} J_\infty =J_{{\textit{KN}}}. \end{aligned}$$

For a discussion of this conservation law in detail see Sect. 2. Using (26), (23), (18) and (15) we finally obtain the dynamical version of the inequality (2)

27 $$\begin{aligned} \frac{Q_\infty ^2+\sqrt{Q_\infty ^4+4J_\infty ^2}}{2} \le M^2. \end{aligned}$$

We emphasize that the inequality (27) holds under the assumption of axial symmetry and electrovacuum in the exterior region of the black hole. In fact, it is known (Huang et al. 2011) that axial symmetry is a necessary condition for (27) to be valid.

We have seen that the two inequalities (2) and (3) extend to the dynamical regime in the forms (21) and (27). These inequalities are global because the mass *M* is the total mass of the spacetime. Whether the quantities in these inequalities can be replaced by an appropriate defined black hole quasilocal mass and angular momentum is unknown.

Penrose argument is remarkable because starting from conjectures (i) and (ii) one is able to deduce inequalities that can be written purely in terms of the initial conditions. That is, the inequalities do not involve the unknown parameters $M_{{\textit{KN}}}$, $Q_{{\textit{KN}}}$, $J_{{\textit{KN}}}$, $A_{{\textit{KN}}}$ of the remainder black hole. A counter example to inequalities (21) or (27) in axial symmetry, will be a counter example of the conjectures (i) or (ii). On the other hand, the proof of such inequalities gives indirect evidences of the validity of the conjectures (i) and (ii). In that sense, the physical heuristic argument is quite strong: in either direction (i.e. if the inequalities are valid or not) it provides highly non-trivial new insight. In contrast, the physical heuristic arguments for the validity of the quasilocal inequality (4) in the dynamical regime are less conclusive.

The argument we present in Sect. 1.1 uses thermodynamics, and hence its validity outside equilibrium is not clear. Nevertheless, the quantities involved in (4) are, as we have seen in Sect. 2, well defined quasilocal quantities in the full dynamical regime (in the case of angular momentum we need the additional assumption of axial symmetry). Consider a stationary black hole that satisfies inequality (4) (we can assume instead that it satisfies the more general version (13), the following argument will be identical). We make a perturbation to this stationary black hole that preserves the charge and angular momentum of the black hole. For example, a vacuum axially symmetric perturbation will have this property. Physically, the stationary black hole will absorb axially symmetric gravitational waves without changing its charge and angular momentum. The area, however, will increase. That means that the same inequality (4) will be satisfied for this dynamical black hole in the future. Slightly more general, if we have a system of multiple black holes such that in the past they can be approximated by isolated stationary black holes and such that the whole spacetime is axially symmetric and electrovacuum, then, by the same argument we expect that the inequality (4) will be satisfied for each individual black hole. Head on collision of black holes is an example of such situation. Hence, by this simple argument, we expect a large class of dynamical black holes for which the inequality (4) is satisfied. However, it is not obvious how to rule out black holes that can not be treated as continuous deformation of stationary black holes (although perhaps such situation does not occur). For those cases, we can argue as follows. If the inequality is not satisfied for a dynamical black hole, then it should be possible to perturb it in the same way as above, increasing the area and preserving the angular momentum and charge. There is in principle no physical restriction to how much the area changes, as long as it increases. Hence, it should be possible to increase the area until the equality in (4) is reached. The arguments presented in Sect. 1.1 suggest that the equality is reached only for extreme black holes. And there are well known physical arguments which suggest that extremal black holes can not be produced in a finite process (Bardeen et al. 1973; Carroll et al. 2009).

Finally, we review the original argument in favor of (4) presented in Dain (2010) (see also Dain 2012). Consider the formula (1) for the horizon area of the Kerr–Newman black holes. From this expression, we can write the mass in terms of the other parameters. Since in axial symmetry we have a well defined quasilocal definition of angular momentum (Sect. 2) we can formally define the quasilocal mass of a black hole by the same expression as the mass for the Kerr–Newman black hole but replacing the parameters by its quasilocal definition, namely

28 $$\begin{aligned} M_{bh}(\mathcal {S})= \sqrt{\frac{A(\mathcal {S})}{16\pi }+ \frac{Q(\mathcal {S})^2}{2}+\frac{\pi (Q(\mathcal {S})^4 +4J(\mathcal {S})^2)}{A(\mathcal {S})}}. \end{aligned}$$

Note that in (28) we have used the total quasilocal angular momentum (i.e. gravitational plus electromagnetic) [see definition (136) in Sect. 2.5]. The relevant question is: does $M_{bh}$ describe the quasilocal mass of a non-stationary axially symmetric black hole? This question is closely related to the validity of the inequality (4) in the dynamical regime. In order to answer it, let us analyze the evolution of $M_{bh}$.

By the area theorem, we know that the horizon area will increase. If we assume axial symmetry and electrovacuum, then the total angular momentum and the charge will be conserved at the quasilocal level as we see in Sect. 2. On physical grounds, one would expect that in this situation the quasilocal mass of the black hole increases with the area, since there is no mechanism at the classical level to extract mass from the black hole. In effect, the only way to extract mass from a black hole is by extracting angular momentum through a Penrose process (Penrose and Floyd 1971; Christodoulou 1970). But angular momentum transfer is forbidden in electrovacuum axial symmetry. Then, one would expect both the area *A* and the quasilocal mass $M_{bh}$ to monotonically increase with time.

Let us take a time derivative of $M_{bh}$ (denoted by a dot). To analyze this, it is illustrative to write down the complete differential, namely the first law of thermodynamics (Bardeen et al. 1973)

29 $$\begin{aligned} \delta M_{bh}= \frac{\kappa }{8 \pi } \delta A + \varOmega _H \delta J + \varPhi _H \delta Q, \end{aligned}$$

where

30 $$\begin{aligned} \kappa= & {} \frac{1}{4 M_{bh}} \left( 1- \left( \frac{4\pi }{A}\right) ^2 (Q^4+4J^2)\right) , \end{aligned}$$

31 $$\begin{aligned} \varOmega _H= & {} \frac{4\pi J}{AM_{bh}}, \quad \varPhi _H= \frac{4\pi (M_{bh}+\sqrt{d}) Q}{A}, \end{aligned}$$

where $M_{bh}$ is given by (27) and *d* [defined in Eq. (1)] is written in terms of *A* and *J* and *Q* as

32 $$\begin{aligned} d= \frac{1}{M_{bh}^2}\left( \frac{A}{16\pi }\right) ^2 \left( 1-(Q^4+4J^2)\left( \frac{4\pi }{A} \right) ^2 \right) ^2. \end{aligned}$$

Under our assumptions, from the formula (28) we obtain

33 $$\begin{aligned} \dot{M}_{bh} = \frac{\kappa }{8\pi }\dot{A} , \end{aligned}$$

were we have used that the total angular momentum *J* and the charge *Q* are conserved. Since, by the area theorem, we have

34 $$\begin{aligned} \dot{A} \ge 0, \end{aligned}$$

the time derivative of $M_{bh}$ will be non-negative (and hence the mass $M_{bh}$ will not decrease with the area) if and only if $\kappa \ge 0$, that is

35 $$\begin{aligned} 4\pi \sqrt{Q^4+4J^2} \le A. \end{aligned}$$

Then, it is natural to conjecture that (35) should be satisfied for any black hole in axially symmetry. If the horizon violates (35) then in the evolution the area would increase but the mass $M_{bh}$ would decrease. This would indicate that the quantity $M_{bh}$ has not the desired physical meaning. Also, a rigidity statement is expected. Namely, the equality in (35) is reached only by the extreme Kerr black hole given by the formula

36 $$\begin{aligned} A = 4\pi \left( \sqrt{Q^4+4J^2 }\right) . \end{aligned}$$

The final picture is that the size of the black hole is bounded from below by the charge and angular momentum, and the minimal size is realized by the extreme Kerr–Newman black hole. This inequality provides a remarkable quasilocal measure of how far a dynamical black hole is from the extreme case, namely an ‘extremality criteria’ in the spirit of Booth and Fairhurst (2008), although restricted only to axial symmetry. Note also that the inequality (35) allows to define, at least formally, the positive surface gravity density (or temperature) of a dynamical black hole by the formula (30) (see Ashtekar and Krishnan 2002, 2003 for a related discussion of the first law in dynamical horizons).

If inequality (35) is true, then we have a non-trivial monotonic quantity (in addition to the black-hole area) $M_{bh}$ in electro-vacuum

37 $$\begin{aligned} \dot{M}_{bh} \ge 0. \end{aligned}$$

There is a different notion of quasilocal energy, known as Hawking energy (Szabados 2004; Bray et al. 2016; Bray and Jauregui 2015; Dafermos 2005), which has monotonic properties under certain energy conditions in various spacetime settings. Whether this property can be used to motivate or prove (4) is an open problem.

It is important to emphasize that the physical arguments presented above in support of (35) are certainly weaker in comparison with the ones behind the Penrose inequalities (21), (8) and (14). A counter example of any of these inequalities in axial symmetry would prove that the standard picture of the gravitational collapse is wrong. On the other hand, a counter example of (35) would only prove that the quasilocal mass (27) is not appropriate to describe the evolution of a non-stationary black hole. One can imagine other expressions for quasilocal mass, maybe more involved, in axial symmetry. On the contrary, reversing the argument, a proof of (35) will certainly suggest that the mass (27) has physical meaning for non-stationary black holes as a natural quasilocal mass (at least in axial symmetry). Also, the inequality (35) provides a non-trivial control of the size of a black hole valid at any time.

### Motivation from Newtonian objects

Geometrical inequalities for ordinary matter fields have gained much interest in the recent years. These inequalities are not expected to arise in Newtonian theory, unless some specific systems or matter fields are considered, which have intrinsic restrictions. With the state of the subject at present, we can not say that geometrical inequalities for objects are produced solely by Einstein equations. Other ingredients must be considered. In particular, one needs an analog of the variational characterization of extreme black holes, that we showed in the previous sections.

What is interesting is that one obtains geometrical inequalities from Newtonian considerations if they are supplemented with a key ingredient from General Relativity, namely, that any speed in the system is not greater than the speed of light. We discuss these inspirational inequalities in what follows.

We first consider the most basic argument in favor of Hoop inequality when we look at an ordinary material object in Newtonian theory. For simplicity, take it to be a spherical, static object of (quasilocal) mass *m* and radius *R*. Then the escape speed from the surface of this object is

38 $$\begin{aligned} v_\mathrm{esc}=\sqrt{\frac{2m}{R}}. \end{aligned}$$

Now, assuming the speed of any particle escaping from the object is not greater than the speed of light, $v_\mathrm{esc}\le 1$, we obtain

39 $$\begin{aligned} m\le \frac{R}{2} \end{aligned}$$

for the object, which agrees with (5).

Now we seek a quasilocal relation between angular momentum and size. We follow Dain (2014b). Consider a Newtonian, spherically symmetric object $\varOmega$ with mass density $\mu$, mass *m* and radius *R*, rotating with speed *v*. Then its angular momentum is

40 $$\begin{aligned} J=\int _{\varOmega }\mu v\rho d^3x \end{aligned}$$

where $\rho$ is the distance to the symmetry axis and $d^3x$ is the flat volume element. Since $\rho \le R$ we obtain

41 $$\begin{aligned} J\le R\int _{\varOmega }\mu v d^3x \end{aligned}$$

Now we take from Einstein theory, the condition that the speed should be smaller than the speed of light, $v\le 1$. This allows to bound the angular momentum as

42 $$\begin{aligned} J\le \frac{R^2}{2} \end{aligned}$$

where we have also used inequality (39) to bound the quasilocal mass $m=\int \mu \,d^3x$.

Finally, we consider a global inequality for Newtonian systems satisfying the condition $v\le 1$. We follow Anglada et al. (2017). Let $\varOmega$ be an ordinary object with mass density $\mu$, quasilocal mass *m*, characteristic radius *R*, equatorial radius $R_c$ (namely $R_c$ is the length, divided by $2\pi$, of the greatest axially symmetric circle on $\partial \varOmega$) and angular momentum *J*. Then we expect that the total energy of the system is a sum of the gravitational and internal energies (included in the term $E_0$) and the rotational kinetic energy

43 $$\begin{aligned} E\approx E_0+\frac{J^2}{2I} \end{aligned}$$

where *I* is the moment of inertia of the object. We bound the euclidean distance $\rho$ from the rotation axis, by the equatorial radius $R_c$, and use inequality (39)

44 $$\begin{aligned} I=\int _\varOmega \mu \rho ^2d^3x\le mR_c^2\le \frac{RR_c^2}{2}. \end{aligned}$$

These considerations give

45 $$\begin{aligned} E\gtrsim E_0+\frac{J^2}{RR_c^2}. \end{aligned}$$

Note that we obtain a lower bound on the total energy of the system in terms of $E_0$, the angular momentum and two measures of size. One coming from the Hoop-like inequality (39) and the other coming from rotation. In other words, *R* measures how localized matter is in $\varOmega$ and $R_c$ measures how distributed matter is with respect to the rotation axis.

As opposed to (39), and (42), which are quasilocal inequalities, (45) is global in nature as it contains the total energy of the system.

What is remarkable is that these informal and naive arguments lead to similar inequalities that can be formally obtained from purely relativistic considerations about ordinary, non-black-hole objects. We review them in Sect. 5.

## Basic definitions

In order to extend inequalities (2)–(4) to dynamical black holes and even to more general situations like ordinary objects we need to introduce these elements with detail, and properly define the physical quantities associated to them $(M,Q, J,A, \text{ etc })$ in the fully dynamical regime.

Black holes are the main type of object we present in this article. That is the setting that originally motivated the study of the geometrical inequalities given in this article and where the most important results have been found so far. However, there are other quasilocal objects that are relevant, namely isoperimetric surfaces and bounded regions representing ordinary objects. We present these objects in Sect. 2.2

On the other hand, the physical quantities studied in this review can be divided in three groups: *local quantities*, *global quantities* and *quasilocal quantities*. Local quantities are tensor fields, global quantities are associated to the whole spacetime and quasilocal quantities are associated with finite regions. We define them in Sects. 2.3, 2.4 and 2.5 respectively.

Before proceeding further with these concepts, we fix the basic notation we use throughout the article.

Let $M$ be a 4-dimensional manifold with metric $g_{\mu \nu }$ [with signature $(-+++)$] and Levi-Civita connection $\nabla _\mu$. In the following, Greek indices $\mu , \nu ,\ldots$ are 4-dimensional, they are raised and lowered with the metric $g_{\mu \nu }$ and its inverse $g^{\mu \nu }$. If, in addition, $g_{\mu \nu }$ satisfies Einstein equations

46 $$\begin{aligned} G_{\mu \nu }\equiv {^4R_{\mu \nu }-}\frac{1}{2} {^4R} g_{\mu \nu }=8\pi T_{\mu \nu }-\varLambda g_{\mu \nu }\qquad \text{ on } M, \end{aligned}$$

where $\varLambda$ is a cosmological constant, $T_{\mu \nu }$ is the energy momentum tensor, $^4R_{\mu \nu }$ is the Ricci tensor of the metric $g_{\mu \nu }$ and ${}^4R$, its scalar curvature, then we call $(M,g_{\mu \nu })$ a spacetime.

Initial conditions for Einstein equations (46) are characterized by an *initial data set* $$(\varSigma , h_{ij}, K_{ij}, \mu , j^i)$$ where $$\varSigma $$ is a connected 3-dimensional manifold, $$h_{ij} $$ a (positive definite) Riemannian metric, $$K_{ij}$$ a symmetric tensor field, $$\mu $$ a scalar field and $$j^i$$ a vector field on $$\varSigma $$. These fields satisfy the constraint equations

47 $$\begin{aligned} D_j K^{ij} - D^i K&= -8\pi j^i \end{aligned}$$

48 $$\begin{aligned} {}^3R - K_{ij} K^{ij}+ K^2&=16\pi \mu \end{aligned}$$

on $$\varSigma $$. Here *D* and $${}^3R$$ are the Levi-Civita connection and scalar curvature associated with $$ {h}_{ij}$$, and $$ K = K_{ij} h^{ij}$$. Latin indices $$i,k,\ldots $$ are 3-dimensional, they are raised and lowered with the metric $$ h_{ij}$$ and its inverse $$ h^{ij}$$.

### Asymptotically flat and cylindrical ends

The initial data models an isolated system when the fields are weak far away from the sources. This physical idea is captured in the following definition of asymptotically flat initial data set. Let $B_R$ be a ball of finite radius *R* in $\mathbb {R}^3$. The exterior region $U=\mathbb {R}^3{\setminus } B_R$ is called an *end*. On *U* we consider Cartesian coordinates $x^i$ with their associated euclidean radius $r=\left( \sum _{i=1}^3 (x^i)^2 \right) ^{1/2}$ and $\delta _{ij}$ to be the euclidean metric components with respect to $x^i$. A 3-dimensional manifold $\varSigma$ is called *Euclidean at infinity*, if there exists a compact subset $\mathcal {K}$ of $\varSigma$ such that $\varSigma {\setminus } \mathcal {K}$ is the disjoint union of a finite number of ends $U_k$. The initial data set $(\varSigma , h_{ij}, K_{ij}, \mu , j^i)$ is called *asymptotically flat* if $\varSigma$ is Euclidean at infinity and at every end the metric $h_{ij}$ and the tensor $K_{ij}$ satisfy the following fall-off conditions

49 $$\begin{aligned} h_{ij}=\delta _{ij} +\hat{h}_{ij}, \quad K_{ij}=O(r^{-2}), \end{aligned}$$

where $\hat{h}_{ij}=O(r^{-1})$, $\partial _k \hat{h}_{ij}=O(r^{-2})$, $\partial _l\partial _k\gamma _{ij}=O(r^{-3})$ and $\partial _k K_{ij}=O(r^{-3})$. These conditions are written in terms of Cartesian coordinates $x^i$ attached at every end $U_k$. Here $\partial _i$ denotes partial derivatives with respect to these coordinates.

The fall-off conditions (49) are far from being the minimal requirements for the validity of the theorems presented in this article. We have chosen these particular fall-off conditions because they are simple to present and they encompass a rich family of physical models. For more refined assumptions we will refer to the original references.

See however, Sect. 2.4 where the stronger fall-off condition (71) on the second fundamental form $K_{ij}$ is imposed. This stronger requirement is necessary to make the integral in the definition of angular momentum converge.

An initial data may have more than one asymptotically flat end, and the asymptotic conditions (49) should hold at each one of these ends.

On the other hand, the initial data may have asymptotically cylindrical ends. They are defined in the following way, extracted from Chruściel et al. (2013), Chruściel and Mazzeo (2015) (see also Dain 2010). An asymptotically cylindrical end of $\varSigma$ is ${\mathbb {R}}^+\times N$ where *N* is a compact 2-manifold where $h_{ij}$ and $K_{ij}$ are conformal to fields having the asymptotic form

50 $$\begin{aligned} {\tilde{h}}=dx^2+{\tilde{h}}^N+{\mathcal {O}}(e^{-\nu x}),\qquad {\tilde{K}}={\tilde{K}}^N+{\mathcal {O}}(e^{-\nu x}) \end{aligned}$$

for some metric $h^N$ on *N*, a symmetric 2-tensor field $K^N$ on *N*, and a positive constant $\nu$.

### Black holes and other objects

#### Black holes

Black holes are global concepts referring to the causal structure of the whole spacetime and therefore, can not be defined in terms of local or quasilocal quantities. This property makes practical applications difficult to study and has led to the development of quasilocal meaningful characterizations of black holes. The intuitive idea that a black hole is a region of spacetime from which no signal can escape is captured by the notion of trapped surface described below. See the articles by Beig and O’Murchadha (1996), Dain (2004), Booth (2005), Jaramillo et al. (2008), Mars (2009), Hayward (2011) and Senovilla (2011), for further references, details and discussions on these quasilocal characterizations.

Consider an oriented spacetime $(M,g_{\mu \nu })$ and a closed, oriented, smooth spacelike 2-surface $\mathcal {S}$ in *M*. Let $\ell ^\mu$ and $k^\nu$ be the null vectors spanning the normal plane to $\mathcal {S}$ and normalized such that $\ell ^\mu k_\mu = -1$ (note that there is a boost rescaling freedom $\ell '^\mu =f \ell ^\mu$, $k'^\mu = f^{-1} k^\mu$). In terms of $\ell ^\mu$ and $k^\mu$, the induced metric and the volume element on $\mathcal {S}$ (written as spacetime projectors) are given by $\gamma _{\mu \nu }=g_{\mu \nu }+\ell _\mu k_\nu +\ell _\nu k_\mu$ and $\epsilon _{\mu \nu }=2^{-1}\epsilon _{\lambda \gamma \mu \nu }\ell ^\lambda k^\gamma$ respectively. The expansions of the null congruences of geodesics with tangent vector fields $\ell ^\mu$ and $k^\mu$ are

51 $$\begin{aligned} \theta _+:=\gamma ^{\mu \nu }\nabla _\mu \ell _\nu , \quad \theta _-:=\gamma ^{\mu \nu }\nabla _\mu k_\nu . \end{aligned}$$

The surface $\mathcal {S}$ is called trapped if $\theta _{\pm }<0$ and *weakly trapped* if $\theta _{\pm }\le 0$. The relevance of trapped surfaces comes from the singularity theorems of Penrose (1965) and Hawking (1971) [see also the review article by Senovilla and Garfinkle (2015)]. Under the Weak Cosmic Censorship Conjecture (see Wald 1999 for details on the conjecture and Christodoulou 1999, 2008; Dafermos 2005 for proofs in spherical symmetry), future trapped surfaces in asymptotically flat initial data evolve into black holes and therefore are fair quasilocal representatives of them. Moreover, the location of the trapped surface is related to the location of the event horizon. This, in particular, is important when analyzing size and shape of a trapped surface as a way to obtain information about the size and shape of a black hole.

In this article we are mainly concerned with two particular cases of trapped surfaces:

- *marginally outer trapped surfaces* (MOTSs), for which $\theta _+=0$ on $\mathcal {S}$,
- *minimal* surfaces for which $\theta _\pm =0$.

MOTS are typically located inside the event horizon in dynamical black hole spacetimes and coincide with compact cross sections of the event horizon in stationary black hole spacetimes (Andersson and Metzger 2009). There is, however an important point that one must keep in mind. This is, even when the trapped surfaces are inside the event horizon, their area need not be smaller than the black hole’s area.

Minimal surfaces have played a key role in the study of geometrical inequalities for black holes in General Relativity since the early days and especially since the proof of the positive mass theorem (Huisken and Ilmanen 2001) . Without mention of null expansions, minimal surfaces are characterized by the vanishing extrinsic curvature when seen as surfaces $\mathcal {S}$ embedded in a 3-dimensional slice $\varSigma$. Note also that if the slice is part of a time symmetric initial data $(\varSigma , h_{ij}, K_{ij}\equiv 0)$, then $\mathcal {S}$ is also a MOTS. Moreover, as we show in Sect. 4.2.5, the variational characterization of stable minimal surfaces is closely related to the one for stable MOTSs. Roughly speaking, stability for minimal surfaces and MOTS (and also for isoperimetric surfaces, see Sects. 2.2.2, 5.2.3) implies a minimization of the area function under certain surface deformations (see Sect.  4.1.1 for precise definitions). The relation between stability of MOTS, black holes and black hole inequalities is discussed by Mars (2014).

With the concept of trapped regions, one can study a (globally hyperbolic) black hole spacetime as the manifold $(M=R\times \varSigma , \,g_{\mu \nu })$ where $\varSigma$ is a spacelike Cauchy surface with trapped inner boundary $\mathcal {S}$. This is the approach taken to study quasilocal Lorentzian inequalities in Sect. 4. Other alternative, mainly used in the study of global and quasilocal Riemannian inequalities is to consider $\varSigma$ as a surface with one asymptotically flat end (representing the region far away from the black holes), and as many extra ends as black holes one wishes to consider (Chruściel 2008). The extra ends will be asymptotically flat if the black holes are subextremal and asymptotically cylindrical if they are extremal black holes. These extra ends are usually called punctures in the numerical community and this type of topology seems to be more appropriate to numerical simulations than 3-surfaces with inner boundaries (Immerman and Baumgarte 2009). So, in a sense, the black holes are represented by non-trivial topology on the initial surface $\varSigma$ (see Gannon 1975; Lee 1976; Meeks et al. 1982; Andersson and Metzger 2009; Eichmair 2007; Andersson et al. 2011; Chruściel et al. 2011; Eichmair et al. 2013).

As an example, consider the Schwarzschild metric is standard coordinates

52 $$\begin{aligned} ds^2=-\left( 1-\frac{2M}{r}\right) dt^2+\left( 1-\frac{2M}{r}\right) ^{-1}dr^2+r^2d\theta ^2+r^2\sin ^2\theta d\phi ^2 \end{aligned}$$

This metric describes a black hole as the manifold $M=R\times \varSigma$ with metric (52), where $\varSigma$ is an asymptotically flat Riemannian manifold with inner boundary $\partial \varSigma =\{r=2M\}$. The inner boundary indicates the location of the event horizon. In this case, the sphere $r=2M$ is a minimal surface and a MOTS. There exists a coordinate system where a doubling of $\varSigma$ is performed. Indeed, make the transformation to the isotropic coordinate ${\bar{r}}$ defined as

53 $$\begin{aligned} {\bar{r}}:=\frac{1}{2}\left[ r-M\pm \sqrt{r(r-2M)}\right] . \end{aligned}$$

Then the transformed metric

54 $$\begin{aligned} ds^2=-\left( \frac{1-M/2{\bar{r}}}{1+M/2{\bar{r}}}\right) dt^2+\left( 1+\frac{M}{{\bar{r}}}\right) ^{4}\left[ d{\bar{r}}^2+{\bar{r}}^2d\theta ^2+ {\bar{r}}^2\sin ^2\theta d\phi ^2\right] \end{aligned}$$

is smooth on the doubled manifold. Note that ${\bar{r}}$ is double valued, it describes two copies of the exterior region of Schwarzschild. The horizon corresponds to the surface ${\bar{r}}=M/2$. This metric is invariant under the inversion through the surface ${\bar{r}}=M/2$. The doubled Riemannian manifold has two asymptotically flat ends at ${\bar{r}}\rightarrow 0$ and ${\bar{r}}\rightarrow \infty$ connected by a minimal surface at ${\bar{r}}=M/2$. In this construction the presence of the black hole is manifested through the extra end at ${\bar{r}}\rightarrow 0$.

#### Isoperimetric surfaces and ordinary objects

As we mention in the introduction, Sect. 1.3, geometrical inequalities for non-black hole objects have gained impetus in recent years.

The first difficulty when studying these systems is the characterization of such ordinary objects. This problem does not appear in the black hole case where there is a well identified surface (the trapped surface) locating the black hole, to which one can associate convenient stability properties.

Away from black holes, one can consider isoperimetric surfaces. These surfaces have been studied within the context of geometrical inequalities in General Relativity, mainly in the context of Penrose inequality (Gibbons 1984, 1997; Malec 1992; Gibbons and Holzegel 2006; Corvino et al. 2007). Isoperimetric surfaces are such that its area is a critical point with respect to nearby surfaces enclosing a given volume. This variational characterization what makes them potentially useful for the study of inequalities.

Ordinary objects, on the other hand, are connected, open, bounded sets with smooth boundary in an initial spacelike hypersurface. Intuitively, an ordinary material object would be the bounded region where smooth matter fields have support. The main difficulty in this case is the lack of a variational characterization, which makes the obtention of geometrical inequalities hard to achieve. In Sect. 5.2 we describe what conditions have been imposed on the objects in order to produce the desired physical-geometrical estimate.

### Local physical quantities

The local physical quantities relevant for General Relativity are shown in Einstein equations (46) and, in particular, in Einstein constraints (47), (48). We focus in this section on the energy momentum tensor $T_{\mu \nu }$ describing matter fields.

It is useful to decompose $T_{\mu \nu }$ into an electromagnetic part and a non-electromagnetic part

55 $$\begin{aligned} T_{\mu \nu }=T^{{\textit{EM}}}_{\mu \nu }+T^M_{\mu \nu }, \end{aligned}$$

where $T^{{\textit{EM}}}_{\mu \nu }$ is the electromagnetic energy momentum tensor given by

56 $$\begin{aligned} T^{{\textit{EM}}}_{\mu \nu }= \frac{1}{4\pi }\left( F_{\mu \lambda } F_\nu {}^{\lambda }-\frac{1}{4}g_{\mu \nu } F_{\lambda \gamma } F^{\lambda \gamma } \right) , \end{aligned}$$

and $F_{\mu \nu }$ is the (antisymmetric) electromagnetic field tensor that satisfies Maxwell equations

57 $$\begin{aligned} \nabla ^\mu F_{\mu \nu }&=-4\pi j^{{\textit{EM}}}_\nu , \end{aligned}$$

58 $$\begin{aligned} \nabla _{[\mu } F_{\nu \alpha ]}&=0. \end{aligned}$$

where $j^{{\textit{EM}}}_\nu$ is the electromagnetic current.

No specific matter model will be used, the only equation that $T_{\mu \nu }$ is required to satisfy is the local conservation law (74)

It is important to emphasize that, unless otherwise stated, the tensors $T^{{\textit{EM}}}_{\mu \nu }$ and $T^M_{\mu \nu }$ are not, individually, divergence free.

We assume that the matter fields satisfy the *dominant energy condition*, that is

59 $$\begin{aligned} T_{\mu \nu } v^\mu w^\nu \ge 0, \end{aligned}$$

for all future-directed causal vectors $v^\mu$ and $w^\nu$. We usually impose this condition also on $T^M_{\mu \nu }$.

Summarizing, the relevant local quantity is the energy momentum tensor $T_{\mu \nu }$, which satisfies the local conditions (74) and (59). Two important particular cases are vacuum $T_{\mu \nu }=0$ and electrovacuum $T^{M}_{\mu \nu }=0$.

### Global physical quantities

Global quantities are associated to isolated systems. An isolated system is an idealization in physics that assumes that the sources are confined to a finite region and the fields are weak far away from the sources. This kind of systems are expected to have finite total energy, linear momentum, angular momentum and charge. In General Relativity there are several ways of defining isolated systems. For our purpose the most appropriate definition is through initial conditions for Einstein equations. Most results concerning global inequalities discussed in this article has been proved, so far, in terms of initial conditions.

Consider an initial data set $(\varSigma , h_{ij}, K_{ij}, \mu , j^i)$ satisfying Einstein constraints (47), (48). If we take the initial data as a spacelike surface in the spacetime, with unit timelike normal $t^\mu$, then the matter fields $\mu$ and $j^i$ are given in terms of the energy momentum tensor by

60 $$\begin{aligned} \mu =T_{\mu \nu } t^\mu t^\nu , \quad j_i =T_{\mu i}t^\mu . \end{aligned}$$

The dominant energy condition (59) implies

61 $$\begin{aligned} \mu ^2 \ge j_ij^i. \end{aligned}$$

The decomposition (55) of the matter fields translates into

62 $$\begin{aligned} \mu =\mu _{{\textit{EM}}}+\mu _M, \quad j_i=j_i^{{\textit{EM}}}+j_i^M, \end{aligned}$$

where we have defined

63 $$\begin{aligned} \mu _{{\textit{EM}}}=\frac{1}{4\pi }\left( E^iE_i+B^iB_i \right) ,\quad j_i^{{\textit{EM}}}=\epsilon _{ijk} E^jB^k, \end{aligned}$$

and the electric field *E* and magnetic field *B* are given by

64 $$\begin{aligned} E_\mu =F_{\mu \nu } t^\nu , \quad B_\mu =- {}^*F_{\mu \nu } t^\nu , \end{aligned}$$

where the dual of $F_{\mu \nu }$ is defined with respect to the volume element $\epsilon _{\mu \nu \lambda \gamma }$ of the metric $g_{\mu \nu }$ by the standard formula

65 $$\begin{aligned} {}^*\alpha _{\mu _1\cdots \mu _{4-p}}=\frac{1}{p!}\alpha ^{\nu _1\cdots \nu _p} \epsilon _{\nu _1\cdots \nu _p \mu _1\cdots \mu _{4-p}}. \end{aligned}$$

The electric charge density $\rho _E$ is defined by

66 $$\begin{aligned} D^i E_i=4\pi \rho _E. \end{aligned}$$

In vacuum we have $\mu =0$, $j^i=0$, and in electrovacuum, $\mu _M=0$, $j^i_M=0$.

For asymptotically flat initial data the expressions for the total energy and linear momentum of the spacetime were presented in Arnowitt et al. (1962) (see also Bartnik 1986; Chruściel 1986) and they are called the ADM energy and linear momentum. They are defined as integrals over 2-spheres $\mathcal {S}_r$ at infinity at every asymptotically flat end by the following formulae

67 $$\begin{aligned} E&=\frac{1}{16\pi }\lim _{r\rightarrow \infty } \oint _{\mathcal {S}_r} \left( \partial _j h_{ij}-\partial _i h_{jj}\right) s^i ds_0\end{aligned}$$

68 $$\begin{aligned} P_i&= \frac{1}{8\pi } \lim _{r\rightarrow \infty } \oint _{\mathcal {S}_r} \left( K_{ik}-Kh_{ik} \right) s^k ds_0, \end{aligned}$$

where $s^i$ is its exterior unit normal and $ds_0$ is the surface element of the 2-sphere with respect to the euclidean metric. The energy *E* and the linear momentum $P_i$ are components of a 4-vector $(E,P_i)$. The total mass of the spacetime is defined by

69 $$\begin{aligned} M=\sqrt{E^2-P^2}, \end{aligned}$$

where we have used the notation $P^2=P_i P_j\delta ^{ij}$.

Let $\beta ^i$ be an infinitesimal generator for rotations with respect to the flat metric associated with the asymptotically flat end *U*, then the angular momentum *J* in the direction of $\beta ^i$ is given by

70 $$\begin{aligned} J_\infty (\beta )=\frac{1}{8\pi } \lim _{r\rightarrow \infty } \oint _{\mathcal {S}_r} (K_{ij} -Kh_{ij})\beta ^i s^j ds_0. \end{aligned}$$

The fall-off conditions (49) are not sufficient to ensure the convergence of the integral (70), extra assumptions are needed. For the results presented in this review which involve the angular momentum $J_\infty$, a stronger fall-off condition on the second fundamental form $K_{ij}$ is imposed

71 $$\begin{aligned} K_{ij}=O(r^{-3}). \end{aligned}$$

In particular this assumption implies that the linear momentum vanishes.

The total electric and magnetic charges are given by Ashtekar et al. (2000b)

72 $$\begin{aligned} Q_{E\infty }= & {} \frac{1}{4\pi } \lim _{r\rightarrow \infty } \oint _{\mathcal {S}_r} E_i s^i ds_0. \end{aligned}$$

73 $$\begin{aligned} Q_{B\infty }= & {} \frac{1}{4\pi } \lim _{r\rightarrow \infty } \oint _{\mathcal {S}_r} B_i s^i ds_0\end{aligned}$$

and we will usually denote them collectively as $Q_\infty$. Note that the magnetic charge does not refer to a magnetic monopole, as it is believed that they do not exist in nature, but it reflects a non-trivial *U*(1) fiber bundle (see Ashtekar and Krishnan 2004 for details).

We emphasize that for every asymptotically flat end $$U_k$$ we have the corresponding, in principle different, quantities $$E_{(k)}$$, $$ P^i_{(k)}$$, $$J^i_{ (k) \infty }$$, $$Q_{(k) \infty }$$.

We use a subscript $\infty$ in the notation for $J_\infty$ and $Q_\infty$, to distinguish them from the quasilocal quantities presented in Sect. 2.5. However, since we will not discuss geometrical inequalities involving quasilocal mass or linear momentum we will not use a subscript $\infty$ in *E* and $P^i$. We expect future extensions of inequalities (2)–(4) in this direction, giving purely quasilocal geometrical inequalities.

### Quasilocal physical quantities

Quasilocal quantities depend on a bounded spacelike 3-dimensional region $\varOmega$, which can be thought as a subset of some initial data $\varOmega \subset \varSigma$. There are two kinds of quasilocal quantities, the first ones depend only on the boundary of the region $\varOmega$, that is a 2-dimensional spacelike closed surface that we will denote by $\mathcal {S}$. The second ones depend also on the interior of $\varOmega$ (for more details on this classification, that is both subtle and important, see Szabados 2004). See also Wieland (2017), Chen et al. (2016), Epp et al. (2013), Tung (2009), Yoon (2004) and Nester et al. (2004). It turns out that for black holes only quasilocal quantities of the first kind are relevant. For objects, quantities of the second kind are also needed. For example, in spherical symmetry the geodesic distance to the center is a relevant quasilocal measure of size. We will present some of these measures with more detail in Sect. 5. Below, we concentrate on quasilocal quantities that depend only on 2-dimensional closed spacelike surfaces $\mathcal {S}$.

On $\mathcal {S}$ we define *intrinsic* and *extrinsic* quantities. The former depend only on the induced Riemannian 2-dimensional metric on the surface that we denote by $q_{ij}$. The extrinsic quantities depend also on the extrinsic curvature of the surface and possible additional fields like the electromagnetic fields. The most important intrinsic quantity is the area $A(\mathcal {S})$ of the surface. For black holes, this is certainly the most relevant intrinsic quantity. But, even for black holes, there exists also other intrinsic quantities that measure the shape of the surface and satisfy geometrical inequalities. We will present them in Sect. 4.2.

#### Conserved quantities

For the discussion of conserved quasilocal quantities, we essentially follow Sect. 2 in Szabados (2004) and Weinstein (1996), see also Dain (2014a).

Consider an arbitrary energy-momentum tensor $T_{\mu \nu }$ which satisfies the conservation equation

74 $$\begin{aligned} \nabla _\mu T^{\mu \nu }=0. \end{aligned}$$

on the curved background $(M,g_{\mu \nu })$ [we are not assuming Einstein equations (46)]. Assume that the spacetime admits a Killing vector field $\eta ^\mu$, that is

75 $$\begin{aligned} \nabla _{(\mu } \eta _{\nu )}=0. \end{aligned}$$

For the present discussion, the vector $\eta ^\mu$ is an arbitrary Killing field, later on we will fix it to be the axial Killing field (i.e. the Killing field associated to axial symmetry). From Eqs. (74) and (75) we deduce that the vector

76 $$\begin{aligned} Z^{\mu }= 8\pi T^{\mu \nu }\eta _\nu , \end{aligned}$$

is divergence free

77 $$\begin{aligned} \nabla _\mu Z^{\mu }=0. \end{aligned}$$

The calculations involved in the definitions of quasilocal quantities require integration over domains with different dimensions and the use of Stokes’ theorem on them. Hence, it is sometimes convenient to use differential forms instead of tensors in order to highlight the geometrical meaning of the integrals. In this section we denote them with boldface.

Let $\varvec{Z}$ be the 1-form defined by (76). Equation (77) is equivalent to

78 $$\begin{aligned} d{}^*\varvec{Z}=0. \end{aligned}$$

Integrating (78) over an orientable, compact but otherwise arbitrary 4-dimensional region of the spacetime and using Stokes’ theorem we obtain the integral form of this conservation law. A particular relevant case is when the 4-dimensional region is a timelike cylinder such that its boundary is formed by the bottom and the top spacelike surfaces $\varOmega _1$ and $\varOmega _2$ and the timelike piece $\mathcal {C}$. For that case, we have

79 $$\begin{aligned} \int _{\varOmega _2 } {}^* \varvec{Z} -\int _{\varOmega _1} {}^* \varvec{Z} =-\int _{\mathcal {C}} {}^* \varvec{Z}, \end{aligned}$$

where the minus sign in the integral over $\varOmega _1$ comes from the choice of the normal. The charge associated to $\varvec{Z}$ of the 3-dimensional spacelike surface $\varOmega$ is defined by

80 $$\begin{aligned} Z(\varOmega )= \int _{\varOmega } {}^* \varvec{Z}, \end{aligned}$$

then we may write Eq. (79) as

81 $$\begin{aligned} Z(\varOmega _2)-Z(\varOmega _1)= -\int _{{\mathcal {C}}} {}^* \varvec{Z}, \end{aligned}$$

Note that the quantities $Z(\varOmega )$ are defined in terms of integrals over 3-dimensional spacelike surfaces. However, Eq. (78) implies, at least locally, that there exists a 2-form ${}^*\varvec{V}$ such that

82 $$\begin{aligned} {}^*\varvec{Z}=d {}^*\varvec{V}. \end{aligned}$$

The 2-form $\varvec{V}$ is called a superpotential for the 3-form ${}^*\varvec{Z}$. We have chosen the dual ${}^*\varvec{V}$ instead of $\varvec{V}$ in the definition (82) in order to make the analogy below, with the Maxwell form ${}^*\varvec{F}$ more transparent. Then, using (82) and Stokes’ theorem once again we have

83 $$\begin{aligned} Z(\varOmega )=\int _{\varOmega }{}^*\varvec{Z}=\int _{\varOmega }d{}^*\varvec{V}=\int _{\partial \varOmega }{}^*\varvec{V} \end{aligned}$$

Denoting by $\mathcal {S}$ the boundary $\partial \varOmega$ we arrive at the conservation law

84 $$\begin{aligned} Z(\mathcal {S}_2)-Z(\mathcal {S}_1)= -\int _{\mathcal {C}} {}^*\varvec{Z}, \end{aligned}$$

where we have defined the quasilocal quantity $Z(\mathcal {S})$ by

85 $$\begin{aligned} Z(\mathcal {S})= \int _{\mathcal {S}} {}^* \varvec{V}. \end{aligned}$$

For example, consider the electromagnetic energy momentum tensor $T^{{\textit{EM}}}$, and let $\eta ^\mu$ be a spacelike Killing vector (for instance, the axial Killing vector). Assume that $\eta ^\mu$ is tangent to $\varOmega$. Then we have a rotation axis $\beta$ and

86 $$\begin{aligned} Z^{{\textit{EM}}}(\varOmega )&= 8\pi \int _{\varOmega } T^{{\textit{EM}}}_{\mu \nu } t^\mu \eta ^\nu \end{aligned}$$

87 $$\begin{aligned}&= 8\pi \int _{\varOmega } E^j B^k \eta ^i \epsilon _{ijk} dv\end{aligned}$$

where $t^\mu$ is the unit vector field normal to $\varOmega$. In Minkowski, a rotation around an arbitrary vector $\beta ^i\not \equiv 0$ is given by

88 $$\begin{aligned} \eta _i =\epsilon _{ijk}\beta ^j x^k, \end{aligned}$$

where $x^i$ are Cartesian coordinates. Then, the expression (86) reduces to

89 $$\begin{aligned} Z^{{\textit{EM}}}(\varOmega ) = 8\pi \int _{\varOmega } E_{[i} B_{j]} x^i \beta ^j. \end{aligned}$$

This is the formula for the angular momentum (in the direction of $\beta$) of the electromagnetic field used in textbooks (see, for example, Jackson 1999; Zangwill 2013).

In Minkowski this construction provides, for each Killing vector field $\eta ^\mu$, the conservation law for all relevant physical quantities associated with the matter field $T^{\mu \nu }$ (i.e. energy, linear momentum, angular momentum).

#### Electromagnetic charge

The most simple and important extrinsic quasilocal quantity on a closed 2-surface $\mathcal {S}$ is the electromagnetic charge $Q(\mathcal {S})$. Its definition and properties serve as model for all the other quasilocal quantities defined on $\mathcal {S}$. Let $\varvec{F}$ be the 2-form corresponding to the electromagnetic tensor $F_{\mu \nu }$, and let ${}^*\varvec{F}$ be its dual. In terms of forms, Maxwell equations (57) are written as

90 $$\begin{aligned} d {}^* \varvec{F}&=4\pi {}^*\varvec{j}^{{\textit{EM}}}, \end{aligned}$$

91 $$\begin{aligned} d \mathbf {F}&=0. \end{aligned}$$

The conservation law for the current $\varvec{j}^{{\textit{EM}}}$ is obtained by taking an exterior derivative to Eq. (90), namely

92 $$\begin{aligned} d {}^*\varvec{j}^{{\textit{EM}}}=0. \end{aligned}$$

Integrating (92) over a 4-dimensional timelike cylinder with boundaries $\varOmega _1$, $\varOmega _2$ and $\mathcal {C}$, as in Sect. 2.5.1, gives

93 $$\begin{aligned} \int _{\varOmega _2 } {}^* \varvec{j}^{{\textit{EM}}} -\int _{\varOmega _1} {}^* \varvec{j}^{{\textit{EM}}} =-\int _{\mathcal {C}} {}^* \varvec{j}^{{\textit{EM}}}. \end{aligned}$$

The electric charge of the 3-dimensional spacelike surface $\varOmega _2$ is defined by

94 $$\begin{aligned} Q_E(\varOmega )= \int _{\varOmega } {}^* \varvec{j}^{{\textit{EM}}}. \end{aligned}$$

Using Eq. (90) in the left-hand side of Eq. (93) we can apply again Stokes’ theorem over the 3-surfaces $\varOmega _1$ and $\varOmega _2$ with boundaries $\mathcal {S}_1$ and $\mathcal {S}_2$ respectively. We obtain

95 $$\begin{aligned} Q_E(\mathcal {S}_2)- Q_E(\mathcal {S}_1)= -\int _{\mathcal {C}} {}^*\varvec{j}^{{\textit{EM}}}, \end{aligned}$$

where now the electric charge $Q(\mathcal {S})$ is defined by the following surface integral over $\mathcal {S}$

96 $$\begin{aligned} Q_E(\mathcal {S})=\frac{1}{4\pi }\int _{\mathcal {S}} {}^* \varvec{F}. \end{aligned}$$

Equation (95) is the conservation law for the electric charge. It depends only on Eq. (92). This equation implies that, at least locally, there exists a 2-form ${}^* \varvec{F}$ such that (90) holds.

In electromagnetism, we start with the field Eqs. (90)–(91) and then we deduce (92) and hence the conservation of $Q_E$.

Similarly, by taking the exterior derivative of (91) one obtains that the magnetic charge

97 $$\begin{aligned} Q_B(\mathcal {S})=\frac{1}{4\pi }\int _{\mathcal {S}} \varvec{F} \end{aligned}$$

is conserved (Ashtekar et al. 2000b), that is

98 $$\begin{aligned} Q_B(\mathcal {S}_1)= Q_B(\mathcal {S}_2). \end{aligned}$$

Equation (98) means that $Q_B$ depends only on the homology class of $\mathcal {S}$. If $\mathcal {S}$ can be shrunk to a point, then $Q_B(\mathcal {S})=0$.

In particular, when $\varvec{j}^{{\textit{EM}}}=0$ in $\varOmega$ the charge has the same value, namely

99 $$\begin{aligned} Q_E(\mathcal {S}_1)= Q_E(\mathcal {S}_2). \end{aligned}$$

That is, when no sources are present the electric charge $Q_E(\mathcal {S})$ also depends only on the homology class of $\mathcal {S}$.

In order to make contact between quantities written in terms of differential forms and other equivalent expressions used in the literature written in terms of tensors it is convenient to choose a tetrad adapted to a closed, oriented, spacelike 2-surface $\mathcal {S}$. Consider null vectors $\ell ^\mu$ and $k^\nu$ spanning the normal plane to $\mathcal {S}$ and normalized as $\ell ^\mu k_\mu = -1$, leaving a (boost) rescaling freedom $\ell '^\mu =f \ell ^\mu$, $k'^\mu = f^{-1} k^\mu$. The induced metric and the volume element on $\mathcal {S}$ (written as spacetime projectors) are given by $q_{\mu \nu }=g_{\mu \nu }+\ell _\mu k_\nu +\ell _\nu k_\mu$ and $\epsilon _{\mu \nu }=2^{-1}\epsilon _{\lambda \gamma \mu \nu }\ell ^\lambda k^\gamma$ respectively. The area measure on $\mathcal {S}$ is denoted by $ds$.

Using tensors and the adapted null vectors $\ell ^\mu$ and $k^\mu$ defined above, the electric and magnetic charges (96), (97) are equivalent to

100 $$\begin{aligned} Q_E(\mathcal {S})= & {} \frac{1}{4\pi }\int _{\mathcal {S}} F_{\mu \nu } \ell ^\mu k^\nu \, ds. \end{aligned}$$

101 $$\begin{aligned} Q_B(\mathcal {S})= & {} \frac{1}{4\pi }\int _{\mathcal {S}} {}^*F_{\mu \nu } \ell ^\mu k^\nu \, ds. \end{aligned}$$

To relate quasilocal quantities with global quantities it is useful to consider 2-surfaces $\mathcal {S}$ that are boundaries of compact subsets $\varOmega$ of the initial data $\varSigma$. Let $t^\mu$ denote the spacetime unit, timelike normal of $\varOmega$. Let $s^\mu$ be the unit, spacelike, normal of $\mathcal {S}$ pointing in the outward direction to $\varOmega$ lying on $\varOmega$. The outgoing and ingoing null geodesics orthogonal to $\mathcal {S}$ defined above are given by $\ell ^\mu =(t^\mu +s^\mu )/\sqrt{2}$ and $k^\mu =(t^\mu -s^\mu )/\sqrt{2}$.

The quasilocal electric and magnetic charges are given by the same expressions (72), (73) but the integrals are taken over the surface $\mathcal {S}$, that is

102 $$\begin{aligned} Q_E(\mathcal {S})= & {} \frac{1}{4\pi } \oint _{\mathcal {S}} E_i s^i ds. \end{aligned}$$

103 $$\begin{aligned} Q_B(\mathcal {S})= & {} \frac{1}{4\pi } \oint _{\mathcal {S}} B_i s^i ds. \end{aligned}$$

In particular, the total charge $Q_\infty$ (that is, the charge contained in $\varSigma$) is obtained as the limit

104 $$\begin{aligned} Q_\infty = \lim _{r\rightarrow \infty } Q(\mathcal {S}_r), \end{aligned}$$

where the sequence of surfaces $\mathcal {S}_r$ are chosen in the same asymptotic end.

#### Angular momentum

To define quasilocal angular momentum in general is a difficult problem (see the review by Szabados 2004). However, for axially symmetric spacetimes there exists a simple and well defined notion of quasilocal angular momentum which was introduced by Komar (1959) (see also Wald 1984). In the literature this definition is usually discussed in vacuum settings, where the angular momentum is conserved. Remarkably, it turns out that the quasilocal inequalities of the form (4) are still valid in the non-vacuum case. The inclusion of matter fields (in particular, electromagnetic fields) presents some peculiarities in the definitions and also in the discussion of the conservation (and non-conservation) of angular momentum.

We have seen in Sect. 2.5.2 that the superpotentials $\varvec{V}$ for electric and magnetic charges are given by $\varvec{V}_E=\varvec{F}/4\pi$ and $\varvec{V}_B={}^*\varvec{F}/4\pi$.

However, there is in general no explicit formula for the superpotential $\varvec{V}_J$ that gives rise to angular momentum, in terms of the fields. For example, consider the electromagnetic tensor $T^{{\textit{EM}}}_{\mu \nu }$. Let $A_\mu$ be the electromagnetic potential defined by

105 $$\begin{aligned} F_{\mu \nu }= \nabla _\mu A_\nu - \nabla _\nu A_\mu , \quad \varvec{F} =d\varvec{A}. \end{aligned}$$

Since $\varvec{V}_J$ is calculated as an integral of $T^{{\textit{EM}}}_{\mu \nu }$ (which involves squares of $F_{\mu \nu }$), a naive counting of derivatives suggests that $\varvec{V}_J$ could be written as products between $F_{\mu \nu }$ and $A_\mu$. However, it appears not to be possible to obtain such explicit expression independent of the solutions.^1^ In order to get such expression we need to impose the solution to be symmetric with respect to the Killing vector $\eta ^\mu$ and also the surface of integration to be tangent to the Killing vector. In the following, we will explicitly find the superpotential $\varvec{V}_J$ for axially symmetric solutions of Maxwell equation. We generalize the discussion presented in Dain (2012) to include a non-zero electromagnetic current $\varvec{j}^{{\textit{EM}}}$ and also we make contact with other equivalent expressions for the quasilocal angular momentum of the electromagnetic field used in the literature.

Denote by $\eta ^\mu$ the Killing field generator of the axial symmetry. The orbits of $\eta ^\mu$ are either points or circles (Beetle and Wilder 2015). The set of point orbits $\varGamma$ is called the axis of symmetry. Assuming that $\varGamma$ is a surface, it can be proven that $\eta ^\mu$ is spacelike in a neighborhood of $\varGamma$ (see Mars and Senovilla 1993). We will further assume that the Killing vector is always spacelike outside $\varGamma$. Note that if this condition is not satisfied then the spacetime will have closed causal curves, in particular it will not be globally hyperbolic. The form $\eta _\mu$ will be denoted by $\varvec{\eta }$, and the square of its norm by $\eta$, namely

106 $$\begin{aligned} \eta =\eta ^\mu \eta _\mu =|\varvec{\eta }|^2. \end{aligned}$$

We have used the notation $\eta ^\mu$ to denote the Killing vector field and $\eta$ to denote the square of its norm to be consistent with the literature. However, to avoid confusions between $\eta ^\mu$ and its square norm $\eta$, we will denote the vector field $\eta ^\mu$ by $\bar{\eta }$ in equations involving differential forms in the index free notation.

We assume that the Maxwell fields are axially symmetric, namely107where  denotes Lie derivative. Consider the 1-forms defined by

108 $$\begin{aligned} \varvec{\alpha }=\varvec{F}(\bar{\eta }) , \quad \varvec{\beta }={}^* \varvec{F}(\bar{\eta }), \end{aligned}$$

where we have used the standard notation $\varvec{F}(\bar{\eta })=F_{\mu \nu }\eta ^\mu$ to denote contractions of forms with vector fields. From Maxwell equations (90)–(91) and the condition (107) we obtain

109 $$\begin{aligned} d \varvec{\alpha } = 0, \quad d \varvec{\beta }= -4\pi {}^* \varvec{j}^{{\textit{EM}}}(\bar{\eta }). \end{aligned}$$

The first equation in (109) implies that, locally, there exists a function $\chi$ such that

110 $$\begin{aligned} \varvec{\alpha }=d \chi . \end{aligned}$$

We calculate the 1-form $\varvec{Z}$ defined in (76) for the electromagnetic tensor $T^{{\textit{EM}}}_{\mu \nu }$ where now $\eta ^\mu$ is the Killing vector field associated to axial symmetry. We denote it again by $\varvec{Z}^{{\textit{EM}}}$ and obtain

111 $$\begin{aligned} \varvec{Z}^{{\textit{EM}}} =2\left( \varvec{F}(\alpha ) -\frac{1}{4}\varvec{\eta }|F|^2\right) . \end{aligned}$$

We want to write the integral of the 3-form ${}^*\varvec{Z}^{{\textit{EM}}}$ as a boundary integral of a 2-form. In order to do that, we use that $\varvec{\eta }$ and $\varvec{ \beta }$ satisfy the following identity

112 $$\begin{aligned} {}^*(\varvec{\eta } \wedge (\varvec{F}(\alpha ))=\varvec{\alpha } \wedge \varvec{ \beta }, \end{aligned}$$

and we also use the following general identity valid for arbitrary 1-forms

113 $$\begin{aligned} {}^*(\varvec{\eta } \wedge \varvec{Z})\wedge \varvec{\eta }=\eta {}^*\varvec{Z} - {}^*\varvec{\eta } ( \varvec{Z}(\bar{\eta })). \end{aligned}$$

Inserting (112) and (113) in (111) we obtain

114 $$\begin{aligned} {}^*\varvec{Z}^{{\textit{EM}}}=2\varvec{\alpha } \wedge \varvec{\beta } \wedge \hat{\varvec{\eta }} + {}^*\hat{\varvec{\eta }} (\varvec{Z}(\bar{\eta })), \end{aligned}$$

where the 1-form $\hat{\varvec{\eta }}$ is defined by

115 $$\begin{aligned} \hat{\varvec{\eta }}= \frac{\varvec{\eta }}{\eta }. \end{aligned}$$

To write the first term in the right-hand side of (114) as the exterior derivative of a 2-form we use the following simple identity

116 $$\begin{aligned} d(\chi \varvec{\beta }\wedge \hat{\varvec{\eta }})=\varvec{\alpha } \wedge \varvec{\beta } \wedge \hat{\varvec{\eta }} +\chi d\varvec{\beta }\wedge \hat{\varvec{\eta }} +\chi \varvec{\beta }\wedge d \hat{\varvec{\eta }}, \end{aligned}$$

where the potential $\chi$ is defined by (110). Putting (116) in (114) we finally obtain

117 $$\begin{aligned} {}^*\varvec{Z}^{{\textit{EM}}}=2 d(\chi \varvec{\beta }\wedge \hat{\varvec{\eta }}) + 8\pi \chi \hat{\varvec{\eta }} \wedge {}^*\varvec{j}^{{\textit{EM}}}(\bar{\eta }) +\chi \varvec{\beta }\wedge d \hat{\varvec{\eta }} + {}^*\hat{\varvec{\eta }} (\varvec{Z}(\bar{\eta })), \end{aligned}$$

where we have used Eq. (109) to replace the term with $d\varvec{\beta }$ by $\varvec{j}^{{\textit{EM}}}$ in (116).

We integrate Eq. (117) over a 3-surface $\varOmega$ tangential to $\eta ^\mu$, with boundary $\mathcal {S}$. The third and the fourth term in (117) do not contribute to the integral because

118 $$\begin{aligned} d \hat{\varvec{\eta }}(\bar{\eta })=0, \quad \varvec{\beta }(\bar{\eta })=0, \end{aligned}$$

and also the restriction of the 3-form ${}^*\hat{\varvec{\eta }}$ to $\varOmega$ is zero. Hence, we obtain the final result

119 $$\begin{aligned} \frac{1}{8\pi } \int _{\varOmega } {}^*\varvec{Z}^{{\textit{EM}}}= -J_{{\textit{EM}}}(\mathcal {S}) + \int _{\varOmega } \chi \hat{\varvec{\eta }} \wedge {}^*\varvec{j}^{{\textit{EM}}}(\bar{\eta }), \end{aligned}$$

where we have defined the quasilocal angular momentum of the electromagnetic field $J_{{\textit{EM}}}(\mathcal {S})$ by

120 $$\begin{aligned} J_{{\textit{EM}}}(\mathcal {S})=- \frac{1}{4\pi } \int _\mathcal {S}\chi \varvec{\beta } \wedge \hat{\varvec{\eta }}. \end{aligned}$$

The 2-form $\chi \varvec{\beta } \wedge \hat{\varvec{\eta }}$ is the superpotential ${}^*\varvec{V}_J$ used in (85). We remark, however, that the expression (120) is valid only for axially symmetric solutions [i.e. we have assumed (107)] and axially symmetric surfaces (i.e. the Killing field $\eta ^\mu$ is tangent to $\mathcal {S}$).

Note that Eq. (119) is valid for non-zero sources $\varvec{j}^{{\textit{EM}}}$. We discuss the case $\varvec{j}^{{\textit{EM}}}=0$, and more generally, the case $\varvec{j}^{{\textit{EM}}}(\bar{\eta })=0$ for axially symmetric initial data in Sects. 3.2.5 and 4.2.5.

To write the angular momentum (120) in terms of the potential $A_\mu$ defined by (105) we use that

121 $$\begin{aligned} \chi = A_\mu \eta ^\mu , \end{aligned}$$

where we have assumed that the potential $A_\mu$ is also axially symmetric, that is122Then inserting (121) in (120) we get

123 $$\begin{aligned} J_{{\textit{EM}}}(\mathcal {S})= \frac{1}{4\pi } \int _\mathcal {S}(A_\lambda \eta ^\lambda ) F_{\mu \nu } l^\mu k^\nu \, ds. \end{aligned}$$

We consider now the angular momentum of the gravitational field. The analog of the electromagnetic current 1-form $\varvec{j}^{{\textit{EM}}}_\mu$ is played by the 1-form $\hat{\varvec{Z}}$ defined by

124 $$\begin{aligned} \hat{\varvec{Z}}\equiv \hat{Z}_\mu ={}^4R_{\mu \nu }\eta ^\nu . \end{aligned}$$

Using the Killing equation for $\eta ^\mu$ we obtain

125 $$\begin{aligned} \nabla _\mu \hat{Z}^{\mu }=0. \end{aligned}$$

This equation is equivalent to

126 $$\begin{aligned} d{}^*\hat{\varvec{Z}}=0. \end{aligned}$$

Hence, we have conservation law for $\hat{\varvec{Z}}$, as we have discussed above for the matter fields and the form $\varvec{Z}$ defined by (76). For $\hat{\varvec{Z}}$, the Komar identity given by (see Wald 1984)

127 $$\begin{aligned} d {}^*d \varvec{\eta }= 2 {}^* \hat{\varvec{Z}}, \end{aligned}$$

provides an explicit formula for the superpotential of $\hat{\varvec{Z}}$. The quasilocal Komar angular momentum is defined by

128 $$\begin{aligned} J_K(\mathcal {S})= \frac{1}{16\pi } \int _\mathcal {S}{}^* d \varvec{\eta }, \end{aligned}$$

where $\mathcal {S}$ is an arbitrary spacelike closed 2-surface. The conservation law for angular momentum in axial symmetry, which is the exact analog to the charge conservation, reads

129 $$\begin{aligned} J(\mathcal {S}_1)- J(\mathcal {S}_2)=\frac{1}{8\pi } \int _{\mathcal {C}} {}^* \hat{\varvec{Z}}. \end{aligned}$$

The right-hand side of this equation represents the change in the angular momentum of the gravitational field which is produced by the left-hand side, namely the angular momentum of the matter fields.

As in the case of the charge, integrating on the spacelike domain $\varOmega$ with boundaries $\mathcal {S}_1$ and $\mathcal {S}_2$, gives

130 $$\begin{aligned} J_K(\mathcal {S}_2)- J_K(\mathcal {S}_1)= -\int _{\varOmega } {}^* \hat{\varvec{Z}}. \end{aligned}$$

In particular, in vacuum we have $\hat{\varvec{Z}}=0$ and hence the angular momentum has the same value on both surfaces, namely

131 $$\begin{aligned} J_K(\mathcal {S}_1)= J_K(\mathcal {S}_2). \end{aligned}$$

That is, for axially symmetric vacuum solutions of Einstein equations the angular momentum $J_K(\mathcal {S})$ depends only on the homology class of $\mathcal {S}$. If $\mathcal {S}$ can be shrunk to a point, then $J_k(\mathcal {S})=0$.

In terms of tensors we have the equivalent expression for the Komar angular momentum

132 $$\begin{aligned} J_K(\mathcal {S})=\frac{1}{16\pi }\int _\mathcal {S}\epsilon _{\mu \nu \lambda \gamma }\nabla ^\lambda \eta ^\gamma \, ds. \end{aligned}$$

Using Einstein equations (46) we can relate the form $\varvec{Z}$ of the matter fields (76) and the form $\hat{\varvec{Z}}$ of the gravitational field

133 $$\begin{aligned} \hat{Z}_\mu =8\pi \left( T_{\mu \nu }\eta ^\nu -\frac{1}{2}T \eta _\mu \right) -\varLambda \eta _\mu , \end{aligned}$$

that is

134 $$\begin{aligned} \hat{\varvec{Z}}= \varvec{Z}-(4\pi T + \varLambda )\varvec{\eta }. \end{aligned}$$

According to (55) and (46) we decompose $\varvec{Z}$ into the electromagnetic part and the non-electromagnetic part

135 $$\begin{aligned} \varvec{Z}=\varvec{Z}^{{\textit{EM}}}+\varvec{Z}^{M}. \end{aligned}$$

The total angular momentum is defined by

136 $$\begin{aligned} J(\mathcal {S})= J_{{\textit{EM}}}(\mathcal {S}) +J_{K}(\mathcal {S}). \end{aligned}$$

It satisfies the conservation law

137 $$\begin{aligned} J(\mathcal {S})= \frac{1}{8\pi } \int _{\varOmega } {}^*\varvec{Z}^{M} +\chi \hat{\varvec{\eta }} \wedge {}^*\varvec{j}^{{\textit{EM}}}(\bar{\eta }). \end{aligned}$$

Note that the cosmological constant term does not contribute because the surface is tangential to $\eta ^\mu$. In terms of tensors, (137) is written as

138 $$\begin{aligned} J(\mathcal {S})= \int _{\varOmega } T^{M}_{\mu \nu } t^\mu \eta ^\nu + A^\mu \eta _\mu j^{{\textit{EM}}}_\nu t^\nu \, dv\end{aligned}$$

There exists a very simple expression for the Komar integral on an initial data, namely

139 $$\begin{aligned} J_K(\mathcal {S})=\frac{1}{8\pi }\int _\mathcal {S}K_{ij} \eta ^i s^j ds. \end{aligned}$$

As in the case of the electric charge, this expression is the quasilocal version of the global expression (70) (recall that $s_i\eta ^i=0$ near infinity). In particular, assuming the fall-off conditions (49), we have

140 $$\begin{aligned} J_\infty (\eta )= \lim _{r\rightarrow \infty } J_K(\mathcal {S}_r)=\lim _{r\rightarrow \infty }J(\mathcal {S}_r), \end{aligned}$$

and

141 $$\begin{aligned} \lim _{r\rightarrow \infty } J_{{\textit{EM}}}(\mathcal {S}_r)=0. \end{aligned}$$

## Global inequalities for black holes

In this section we present results involving the total mass of a black hole and some of its quasilocal physical parameters like the electromagnetic charge and angular momentum. We group the results into two sections. The first group, in Sect. 3.1 refers to inequalities involving total mass and electromagnetic charge, with zero angular momentum. The second group incorporates angular momentum. As we discuss in Sect. 2, in order to have a well defined conserved angular momentum, axial symmetry is required, whereas the pure charge case needs no symmetry at all to be well formulated. This difference makes the techniques used to solve the problems very different as we discuss below. Before going into the details, we want to make two remarks.

*Settings* The geometrical inequalities presented in this section are proven for a set of initial data $(\varSigma , h_{ij}, K_{ij})$ for Einstein equations. Evolution arguments are not considered. Moreover, Einstein constraint equations are used in a crucial way. The initial surface has an asymptotically flat end, where the ADM mass is computed. Also, it may have an inner boundary, connected or not, or be complete, with at least one more end. These features capture the presence of the black hole (see Sect. 2.2). In the introduction, Sect. 1.2, we give arguments indicating that the global black hole inequalities are valid for all times if they are valid at some initial time.

*Inequality producer* We wish to mention here the features in the systems considered that ultimately produce the inequalities. Note that we are not thinking about the general qualities of systems in the theory that allow such inequalities (this was discussed at the beginning of the introduction). But we wonder about the underlying mathematical hypothesis or condition on the initial data $(\varSigma , h_{ij}, K_{ij})$ that translate into the desired relation between the physical and geometrical parameters. Interestingly, in order to derive the global inequalities presented in this section, the only requirement are certain energy conditions and the presence of at least two ends on $\varSigma$ if $\varSigma$ has no inner boundary (one of which needs to be asymptotically flat to have a well defined ADM mass). However, as we present in Sect. 3.1, the mass–charge inequality is also proven when $\varSigma$ is an asymptotically flat manifold with inner trapped boundary. This is in contrast with the Penrose inequality (Mars 2009), which is also global, but where the area of a closed 2-surface is included explicitly into the estimate and therefore, extra assumptions on such 2-surface must be imposed.

### Mass–charge

The mass–charge inequality arises as a way to refine the positive mass theorem (Schoen and Yau 1979, 2017, 1981; Witten 1981) and to give a strictly positive lower bound for the total mass in the Einstein–Maxwell theory.

#### Results

Below is the main theorem showing this result. Its proof is greatly due to Gibbons and Hull (1982), and to Gibbons et al. (1983), but many authors have contributed to the final version. We describe their particular input after the statement.

##### Theorem 1

Let $(\varSigma , h_{ij}, K_{ij}, E^i, B^i, \mu _M, j^i_M)$ be a strongly asymptotically flat initial data for the Einstein–Maxwell equations, with $(\varSigma , h_{ij})$ complete or having a weakly outer trapped inner boundary. Assume that matter fields satisfy the energy condition

142 $$\begin{aligned} \rho _{{\textit{EM}}}\le \sqrt{\mu _M^2-|j_M|^2}. \end{aligned}$$

Then, on every end

143 $$\begin{aligned} |Q_\infty | \le M , \end{aligned}$$

where *M* and $Q_\infty =\sqrt{Q_{E,\infty }^2+Q_{B,\infty }^2}$ are computed at the same end. Moreover

(i) if the equality in (143) is attained then the associated spacetime is, locally, an Israel–Wilson–Perjés metric.
(ii) if the initial data are maximal (i.e. $K=0$) and electro-vacuum, then the equality in (143) holds if and only if the data set arises from the Majumdar–Papapetrou spacetime.
(iii) if the Dirac–Jang equations have an appropriate solution then the equality in (143) holds if and only if the data set arises from the Majumdar–Papapetrou spacetime.

As we mention above, (143) is proven in Gibbons and Hull (1982) and Gibbons et al. (1983). They use spinorial arguments similar to ones used in Witten’s proof of the positive mass theorem (Witten 1981). Gibbons and Hull (1982) shows that the equality in (143) holds if and only if there exists a super covariantly constant spinor, and the Israel–Wilson–Perjés and Majumdar–Papapetrou metrics are discussed in this context.

Tod (1983) (see also Herzlich 1998; Horowitz 1984) addresses the rigidity statement (i) by finding all smooth spacetimes admitting such super covariantly constant spinors. They are gravitational and electromagnetic plane waves possibly with dust, and metrics describing charged rotating dust. Particular cases of the latter are the Israel–Wilson–Perjés metrics, some of the Bonnor metrics and the Majumdar–Papapetrou metric.

Chruściel et al. (2006b) prove the rigidity statement (ii) by showing that under certain conditions, the Israel–Wilson–Perjés metrics are of Majumdar–Papapetrou class.

Bartnik and Chruściel (2005) generalize the proof of (143) to include low differentiable metrics, namely $h_{ij}\in H^2_{loc}$ and $K_{ij}, E^i, B^i \in H^1_{loc}$. However, the equality is left open, as the classification of metrics admitting super covariantly constant spinors of Tod (1983) does not apply in the rigidity case.

The rigidity statement (iii) is proven by Khuri and Weinstein (2013) assuming that a system of equations, namely the Dirac–Jang equations, has appropriate solutions. They also assume what they call *the charged dominant condition*, which reads $\rho _{{\textit{EM}}}\ge \mu _M-|j_M|$ and is stronger than condition (142).

A related mass–charge inequality was proved by Moreschi and Sparling (1984) with similar spinorial techniques, and also later by Bartnik and Chruściel (2005). More precisely, if instead of (142), matter fields satisfy

144 $$\begin{aligned} \alpha \rho _{{\textit{EM}}}\le \sqrt{\mu _M^2-|j_M|^2} \end{aligned}$$

for some $\alpha \in (0,1]$, then the inequality $|Q_\infty | \le \alpha M$ holds. Note that when $\alpha =1$ it reduces to (142). This result is relevant for ordinary matter (see the first remark below).

#### Discussion

A few comments about the hypotheses and applications follow.

The energy condition (142) can be interpreted as a local version of the global inequality (143) . Namely, write (143) as

145 $$\begin{aligned} \sqrt{P^2+ Q^2_\infty }\le E, \end{aligned}$$

then *P*, $Q_\infty$ and *E* are the global quantities corresponding to the local quantities $j_M$, $\rho _E$ and $\mu _M$ in (142). In that sense, condition (142) looks rather natural. However it is important to recall that ordinary charged matter can violate this condition and, of course, also the global inequality (143) (see the discussion in Gibbons and Hull 1982; Horowitz 1984; Moreschi and Sparling 1984; Dain 2012). The ultimate reason for that is that the mass charge relation of the electron violates the inequality (143) for several orders of magnitude. In fact, the local condition (142) allows only matter fields with very small amount of electric charge. One way to avoid this limitation is to relax the condition on matter fields, for instance, by asking (144). Then the inequality obtained has a much wider applicability. In the electro-vacuum case we have $\rho _E=0$, then condition (142) reduces to the dominant energy condition for the non-electromagnetic matter fields.

The manifold $\varSigma$ can have multiple asymptotic ends, on different ends the value of the quantities $E,P, Q_\infty$ are different and the inequality (143) holds on every end. Also, the theorem admits a manifold with an inner boundary given by the 2-surface $\mathcal {S}$. Consider the simplest case, where the manifold $\varSigma$ is $\mathbb {R}^3$ (which, of course, means that it has only one asymptotic end and no inner boundary). For that case, in order to have non-zero $Q_\infty$ we need to have charged matter in the interior, that is $\rho _E \ne 0$. The value of $Q_\infty$ represents the total charge of the spacetime, it will be in general different than the value of the charge calculated for an arbitrary surface $Q(\mathcal {S})$ in the interior. An important spacetime that satisfies these conditions is the electrically counterpoised dust studied by Bonnor (see Bonnor 1980, 1998 and reference therein). These are explicit solutions that describe static configurations of charged dust with $\mu _M=|\rho _E|$, and hence the gravitational attraction is exactly balanced by the electric repulsion. The shape of the configuration can be arbitrary and does not need to have any spatial symmetry. These spacetimes achieve the equality $M=|Q_\infty |$ in (143).

If we assume electro-vacuum, in order to have a non-zero $Q_\infty$ we need to allow either a non-trivial topology with multiple asymptotic ends or a non-trivial inner boundary $\mathcal {S}$. The Reissner–Nordström black hole initial data are the model example of both cases: either we consider them as a complete manifold with two asymptotically flat ends or as a manifold with inner boundary $\mathcal {S}$ at the black hole horizon which is weakly outer trapped. The equality $M=|Q_\infty |$ is achieved by the extreme Reissner–Nordström black hole, which has one asymptotically flat end and one cylindrical end. On the other hand, initial data of the super extreme Reissner–Nordström metric does not satisfy the hypotheses since the Riemannian manifold $(\varSigma ,h_{ij})$ is not complete and does not have any weakly outer trapped 2-surface.

The Majumdar–Papapetrou spacetime also satisfies the hypotheses of Theorem 1. The initial data has only one asymptotically flat end and an arbitrary number of extra cylindrical ends. This spacetime describes the equilibrium configuration of multiple extreme black holes for which the gravitational attraction is balanced by the electric repulsion. It satisfies $M=|Q_\infty |$ (see Hartle and Hawking 1972 for a discussion of these spacetimes). The extreme Reissner–Nordström black hole is a particular case of Majumdar–Papapetrou with only one cylindrical end.

The Israel–Wilson–Perjés metrics are characterized by the existence of a ‘super-covariantly constant’ spinor field (for details about this metrics see Tod 1983; Chruściel et al. 2006b and references therein). Example of this class of metrics are the above mentioned electrically counterpoised dust and the Majumdar–Papapetrou metrics. We emphasize that the equality in (143) can be achieved by a non-electro-vacuum solution. However, for the electro-vacuum case a stronger result is given in statement (i).

### Mass–angular momentum–charge

The inclusion of the angular momentum in the inequality (3) involves completely different techniques as the one used in Theorem 1. In particular no spinorial proof of this inequality with angular momentum is available so far (see however Zhang 1999, where a related inequality is proven using spinors).

#### Results

Below is the mass–angular momentum–charge theorem, the proof in vacuum is mainly due to Dain (2008). Some of the later generalizations and refinements by other authors are included in the statement and discussed after it.

##### Theorem 2

Let $(\varSigma , h_{ij}, K_{ij}, E^i, B^i)$ be an electrovacuum, axially symmetric, maximal, initial data set with two asymptotic ends. One end is asymptotically flat, where the fall-off condition (25) is assumed for the second fundamental form. The other end is asymptotically flat or cylindrical.

Then, the following inequality holds at the asymptotically flat end

146 $$\begin{aligned} \frac{Q_\infty ^2+\sqrt{Q_\infty ^4+4J_\infty ^2}}{2} \le M^2. \end{aligned}$$

The equality in (146) holds if and only if the initial data corresponds to the extreme Kerr–Newman black hole.

The proof of the inequality (146) was provided by Dain in a series of articles (Dain 2006a, b, c), which end up in the global proof given in Dain (2008). There it is shown that (146) holds in vacuum and for a class of axially symmetric black hole initial data known as Brill data. The argument exploits the relation between a certain mass functional $\mathcal {M}(\eta ,\omega )$ and the energy for harmonic maps from $\mathbb {R}^3$ to the Hyperbolic plane (Carter 1973; Ernst 1968) ($\eta$ and $\omega$ are the norm and twist potential of the axial Killing vector). See Sect. 3.2.5 for the definition and properties of $\mathcal {M}$ and details about the proof of Theorem 2.

Along the same lines, Chruściel (2008) and Chruściel et al. (2008) give a simpler proof of the theorem and avoid some technical assumptions on Dain’s proof but consider a class of axially symmetric initial data that do not contain the limit case in vacuum, namely, extreme Kerr. They allow positive matter density that does not enter explicitly into the inequality. Moreover, they assume the existence of a twist potential $\omega$ which is known to exist in vacuum.

Electromagnetic charge is brought into the inequality by Chruściel and Lopes Costa (2009) and Costa (2010). In particular, in Costa (2010) an appropriate potential, related to the twist potential for the axial Killing vector is shown to exist. This allows the definition of a new mass function $\mathcal {M}(\eta ,\omega ,\psi ,\chi )$, where $\eta$ and $\omega$ are as before and $\psi$, $\chi$ are the electromagnetic potentials (see Sect. 3.2.5). As in Chruściel et al. (2008), the initial data considered does not include extreme Kerr–Newman and therefore, the rigidity statement can not be analyzed.

Schoen and Zhou (2013) make several improvements on the assumptions, they give a relevant lower bound for the difference between general data and the extreme Kerr–Newman data and they prove the rigidity statement in the charged case.

In Theorem 2 the data are assumed to be maximal. For the case with no charge, the maximal condition is relaxed to a small trace case assumption by Zhou (2012).

The maximality condition is replaced in Cha and Khuri (2014, 2015) by the assumption that a system of equations has appropriate solutions. Also, non-electromagnetic matter fields $\mu _M,j_M$ that satisfy the charged dominant energy condition $\mu _M\ge |j_M|$ are included in the hypotheses. In these articles a deformation procedure of the initial data is constructed that provides a natural and clean way to automatically generalize geometrical inequalities proved in the maximal case.

The key role that extreme Kerr–Newman black holes play in giving the limit values for inequality (146) (and, as we will see later, in the quasiliocal inequalities for black holes) is confirmed and reinforced by the gedanken experiments performed by Sorce and Wald in Sorce and Wald (2017). There it is proven that an extremal Kerr–Newman black hole can not be over-charged or over-spun.

#### Discussion

Before presenting related results to Theorem 2, a few remarks are in order.

We begin by comparing Theorem 2 and the pure charge Theorem 1. In Theorem 2 axial symmetry is assumed globally on the initial data, in contrast with Theorem 1 where there are no symmetry assumptions. As we have seen in Sect. 1.2, on physical grounds, the inequality (146) is not expected to hold for non-axially symmetric data. General families of non-axially symmetric counter examples of the inequality (146) have been constructed by Huang et al. (2011) for pure vacuum and complete manifolds.

In Theorem 2 electrovacuum is assumed. It is conceivable that, using similar techniques as in the current proof of this theorem, the electrovacuum assumption can be slightly relaxed by assuming, in analogy with the Assumption (142) in Theorem 1, that the matter fields have small angular momentum and charge. We expect that this assumption will be rather unphysical, since ordinary rotating matter can easily violate the inequality (146). It is however interesting that in Theorem 1 a rigidity statement is obtained even in the non-electrovacuum case. It is not known whether an analogous rigidity result holds for axially symmetric matter fields with non-trivial angular momentum in which the equality in (146) is achieved. Note however, that the rigidity statement in Theorem 1 depends strongly on the spinorial proof. It appears to be unlikely that a generalization of this rigidity result holds for the case of angular momentum.

On the other hand, we expect that the dominant energy condition should be required, as there are numerical examples by Bode et al. (2011) of spinning black holes with matter violating the null (and dominant) energy condition, with $J\ge M^2$.

No inner boundary is allowed in Theorem 2. The inclusion of an inner boundary (which presumably should be a weakly trapped surface as in Theorem 1) appears to be a difficult and a relevant problem. An inner boundary requires appropriate boundary conditions for the variational problem used in the proof of Theorem 2. The results presented by Gibbons and Holzegel (2006) and Chruściel and Nguyen (2011) contribute in this direction, but so far the problem remains open.

Since electrovacuum is assumed, in order to have non-zero charge and angular momentum the manifold should have a non-trivial topology. In Theorem 2 a particular geometry is assumed: manifolds with two ends. This is certainly the stronger restriction of this theorem. Let us discuss this point in detail.

The model initial data set that satisfies all the hypotheses of Theorem 2 is a slice $t=constant$ in the standard Boyer–Lindquist coordinates of the Kerr–Newman black hole. In the non-extreme case these initial data have two asymptotically flat ends, where the standard fall-off conditions (49) are satisfied, plus the stronger fall-off condition (71) of the second fundamental form. However, in the extreme case, the geometry of the initial data changes: one end is cylindrical and the other is asymptotically flat. That is, in order to include the extreme case more general fall-off conditions need to be allowed on one of the ends.

#### Multiple black holes

For multiple ends the problem is open. There exist however the following very interesting results. In order to describe them, we need to highlight some properties of the mass functional $\mathcal {M}$ (see Sect. 3.2.5 for more details). This functional represents a lower bound for the mass. Moreover, the global minimum of this functional (under appropriate boundary conditions which preserve the angular momentum) is achieved by a harmonic map with prescribed singularities. This is the main strategy in the proofs of all the previous theorems which are valid for two asymptotic ends. Remarkably enough, Chruściel et al. (2008) prove the existence and uniqueness of this singular harmonic map for manifolds with an arbitrary number of asymptotic ends, and then, as a corollary they prove the following result

##### Theorem 3

Consider an axially symmetric, vacuum asymptotically flat and maximal initial data with *N* asymptotic ends. Denote by $M_i$, $J_i$ $(i=1,\ldots N)$ the mass and angular momentum of the end *i*. Take an arbitrary end (say 1), then the mass at this end satisfies the inequality

147 $$\begin{aligned} \mathcal {M}(J_2,\ldots , J_N) \le M_1 , \end{aligned}$$

where $\mathcal {M}(J_2,\ldots , J_N)$ denotes the numerical value of the mass functional $\mathcal {M}$ evaluated at the corresponding harmonic map.

This theorem reduces the proof of the inequality with multiples ends to compute the value of the mass functional on the corresponding harmonic map.

 Khuri and Weinstein (2016) extend the result by Chruściel et al. (2008) in that electromagnetic charge is included and weaker fall-off conditions are assumed on the extrinsic curvature. Also, the rigidity statement is proven.

Some numerical calculations have been made to get insight about the value $\mathcal {M}(J_2,\ldots , J_N)$. Dain and Ortiz (2009) perform numerical calculations of the mass functional mentioned above (see Sect. 3.2.5) and find evidence for the validity of an intermediate inequality of the form $\mathcal {M}\ge |J_1|$, where $J_1$ is the angular momentum of a system of two Kerr black holes with positive individual masses, computed at the end 1 in the notation of Theorem 3. Following this result, Cabrera-Munguia et al. (2010) work on the Tomimatsu and Dietz–Hoenselaers solution describing two Kerr black holes, one of which has negative mass. They find that there is a rank in the parameters of the individual black holes such that the total mass is smaller than the total angular momentum, that is $M^2{<}|J_1|$ and therefore $\mathcal {M}^2{<}|J_1|$, which opposes to Dain and Ortiz result. These are intesting results that open up the questions of what the reasonable hypotheses on the multiple black hole system should be and of what the precise form the inequality should have. Since the Tomimatsu and Dietz–Hoenselaers solution has a naked singularity, one might expect that inequality $M^2\ge |J_1|$ holds for regular multiple black hole solutions. But on the other hand, there is the possibility of conjecturing and proving a different inequality involving not only ADM mass and angular momentum (whether total or individual), but also other phyisical parameters like separation distance between black holes or some other properties of the system. This is certainly an interesting open problem.

#### Non-asymptotically flat manifolds

Recently, global inequalities for asymptotically hyperboloidal initial data have started to be explored. Cha et al. (2016) generalize a procedure used by Schoen and Yau (1981) which consists in a deformation that transforms an asymptotically hyperboloidal structure into an asymptotically flat one. By doing this, they are able to use the geometrical inequalities known to hold in the asymptotically flat case, to prove them in the hyperboloidal case. More precisely, they consider a smooth, simply connected, axially symmetric initial data satisfying the charged dominant energy condition and the matter condition $\mu _{M}\ge |j_{M}|$ and the matter condition $j_M^i\eta _i=0$. The data is assumed to have two ends, one asymptotically hyperboloidal and the other either asymptotically flat or asymptotically cylindrical. If certain system of equations, consisting of a Jang-like equation, and two extra equations on the deformed data, admits a smooth solution with prescribed asymptotics, then inequality (146) holds. Moreover, if equality is attained, then the initial data arise from an embedding into the extreme Kerr–Newman spacetime. This result effectively reduces the proof of the geometrical inequality to proving existence of solutions to certain equations with appropriate fall-off conditions.

Cha and Khuri (2017) apply the same arguments to obtain an identical global inequality to (146) in the case that the initial data has two ends, one AdS hyperbolic and the other either asymptotically AdS hyperbolic or asymptotically cylindrical. The cosmological constant is not, however, explicitly included into the inequality. See Sect. 3.3 for a conjectured global inequality including cosmological constant.

#### The mass functional $\mathcal {M}$

We present in this section the main arguments behind the mass–angular momentum inequalities (146) and (147). They are heavily based on a certain mass functional $\mathcal {M}$ and its relation with the energy of harmonic maps. For simplicity we assume electrovacuum and maximality to present the basic properties of $\mathcal {M}$ that are crucial for proving (146). We also assume only two ends on the initial data.

The proof consists of two steps. The first one is to prove $m\ge \mathcal {M}$ for the given black hole initial data, using the Hamiltonian equation and some energy conditions. The second step is to prove that extreme Kerr–Newman black hole with the same angular momentum as the given data, is a minimizer for $\mathcal {M}$, that is $\mathcal {M}\ge \mathcal {M}|_{\text{ extr } \text{ Kerr--Newman }}$ using known results on harmonic maps with prescribed boundary conditions. Let us see this in more detail.

**Step 1.** $$m\ge \mathcal {M}$$

Consider an asymptotically flat, axially symmetric, maximal initial data set $(\varSigma , h_{ij}, K_{ij}, E^i, B^i)$. This means that $K_{ij}h^{ij}=0$ and that there exists a rotational Killing vector field $\eta ^i$ such that148where  is the Lie derivative.

Assuming $j^{{\textit{EM}}}_i\eta ^i=0$, the non-trivial constraint equation reads

149 $$\begin{aligned} R=K_{ij}K^{ij}+16\pi (E_iE^i+B_iB^i) \end{aligned}$$

and the Maxwell constraints without sources are

150 $$\begin{aligned} dF=0,\quad d {}^*F=0 \end{aligned}$$

Due to axial symmetry, there exists a coordinate system such that the metric $h_{ij}$ can be written in the form

151 $$\begin{aligned} h=e^{\sigma +2q}(d\rho ^2+dz^2)+\rho ^2e^{\sigma }(d\phi +\rho A_\rho d\rho +A_zdz)^2 , \end{aligned}$$

where the functions $\sigma , q$ and $A_\rho , A_z$ depend only on $\rho ,z$. See the article by Chruściel (2008), where a careful constructive proof of the existence of such coordinate system is done. The two ends correspond to the regions $r=\sqrt{\rho ^2+z^2}\rightarrow \infty$, which is an asymptotically flat end, and $r\rightarrow 0$, which is either asymptotically flat or cylindrical.

We follow Costa (2010) to introduce the following 3-dimensional potentials. Let $\psi$ be the electric potential (compare with the 4-dimensional potential $\psi$ presented in Sect. 2.5) and $\chi$, the magnetic potential given by

152 $$\begin{aligned} d\chi :=F(\bar{\eta }),\qquad d\psi :={}^*F(\bar{\eta }) , \end{aligned}$$

where $[F(\bar{\eta })]_i=F_{ij}\eta ^j$. We also introduce the potential $\omega$ as given by

153 $$\begin{aligned} d\omega :=K(\bar{\eta })\wedge \eta -\chi d\psi +\psi d\chi , \end{aligned}$$

where, similarly, $$[K(\bar{\eta })]_i=K_{ij}\eta ^j$$.

These potentials have two properties that make them highly suited for the problem at hand. The first one is that $\psi , \chi$ and $\omega$ are constant on each connected component of the symmetry axis $\varGamma$. This, in particular, gives a close and simple relation between these potentials and the physical quantities $Q_E$, $Q_B$, $J_\infty$:

154 $$\begin{aligned} Q_{E\infty }=\frac{\psi _+-\psi _-}{2},\quad Q_{B\infty }=\frac{\chi _+-\chi _-}{2},\quad J_\infty =\frac{\omega _+-\omega _-}{8} \, \end{aligned}$$

where the subindex $+$ and − on a quantity *f* indicate the constant values of the function on each connected component of $\varGamma$, namely: $f_+=f(\rho =0,z>0)$ and $f_-=f(\rho =0,z<0)$.

The second property is that they allow us to write the following important expressions

155 $$\begin{aligned} K_{ij}K^{ij}\ge 2\frac{e^{-3\sigma -2q}}{\rho ^4}(\partial \omega +\chi \partial \psi -\psi \partial \chi )^2 \end{aligned}$$

and

156 $$\begin{aligned} E_iE^i+B_iB^i\ge \frac{e^{-2(\sigma +q)}}{\rho ^2}\left[ (\partial \psi )^2 +(\partial \chi )^2\right] , \end{aligned}$$

where in the left-hand sides of (155) and (156), indices are moved with the metric $h_{ij}$ and the right-hand sides involve square gradients with respect to the flat 2-dimensional metric $d\rho ^2+dz^2$.

Also, the curvature scalar *R* of the metric $h_{ij}$ is bounded as

157 $$\begin{aligned} -\frac{1}{8}Re^{\sigma +2q}\ge \frac{1}{4}\varDelta \sigma +\frac{1}{16}(\partial \sigma )^2+\frac{1}{4}\varDelta _2q , \end{aligned}$$

where $\varDelta$ is the flat Laplacian in 3-dimensions, $(\partial \sigma )^2=\frac{1}{\rho ^2}(\partial _\rho \sigma )^2 +(\partial _z\sigma )^2$ and $\varDelta _2:=\partial ^2_\rho +\partial ^2_z$.

Now we integrate the Hamiltonian equation (149) on ${\mathbb {R}}^3$ with the flat volume element $d^3x$ and use the bounds (155), (156) and (157) to obtain

158 $$\begin{aligned} m\ge \frac{1}{32\pi }\int _{{\mathbb {R}}^3}(\partial \sigma )^2+ 4\frac{(\partial \omega +\chi \partial \psi -\psi \partial \chi )^2}{\eta ^2} +4\frac{(\partial \psi )^2+ (\partial \chi )^2}{\eta }\;d^3x , \end{aligned}$$

where we have used the expression of the ADM mass computed from (69) for the particular metric (151)

159 $$\begin{aligned} m=-\frac{1}{8\pi }\int \varDelta \sigma \; d^3x , \end{aligned}$$

the explicit square norm of the axial Killing vector, $\eta =e^\sigma \rho ^2$, and also the asymptotically flat fall-off conditions at infinity and the regularity at the axis $q|_{\varGamma }=0$ to discard the term with $\varDelta _2q$.

Defining the right-hand side of (158) as the mass functional $\mathcal {M}$

160 $$\begin{aligned} \mathcal {M}:=\frac{1}{32\pi }\int _{{\mathbb {R}}^3}(\partial \sigma )^2+ 4\frac{(\partial \omega +\chi \partial \psi -\psi \partial \chi )^2}{\eta ^2} +4\frac{(\partial \psi )^2+ (\partial \chi )^2}{\eta }\;d^3x , \end{aligned}$$

we obtain the desired inequality $m\ge \mathcal {M}$.

**Step 2.** $$\mathcal {M}\ge \mathcal {M}|_{{\text{ extr } \text{ Kerr--Newman }}}$$

We start by restricting the integral in the definition of $\mathcal {M}$ to an open set $\varOmega \subset {\mathbb {R}}^3$, and denoting the corresponding functional as $\mathcal {M}_\varOmega$. We also write it fully in terms of $\eta$ by taking into account that

161 $$\begin{aligned} (\partial \sigma )^2=\left( \frac{\partial \eta }{\eta }-2\partial \ln \rho \right) ^2=\left( \frac{\partial \eta }{\eta }\right) ^2+ 4\partial \ln \rho \partial \left( \ln \eta -\ln \rho \right) . \end{aligned}$$

We obtain

162 $$\begin{aligned} \mathcal {M}_\varOmega= & {} \frac{1}{32\pi }\int _{\varOmega }\frac{(\partial \eta )^2}{\eta ^2}+ 4\frac{(\partial \omega +\chi \partial \psi -\psi \partial \chi )^2}{\eta ^2} +4\frac{(\partial \psi )^2+ (\partial \chi )^2}{\eta }\;d^3x\nonumber \\ \end{aligned}$$

163 $$\begin{aligned}&\,+\frac{1}{8\pi }\int _{\varOmega }\partial \ln \rho \partial \left( \ln \eta -\ln \rho \right) \;d^3x . \end{aligned}$$

We integrate by parts the last integral and use that $\ln \rho$ is harmonic ($\varDelta \ln \rho =0$) to obtain

164 $$\begin{aligned} \mathcal {M}_\varOmega= & {} \frac{1}{32\pi }\int _{\varOmega }\frac{(\partial \eta )^2}{\eta ^2}+ 4\frac{(\partial \omega +\chi \partial \psi -\psi \partial \chi )^2}{\eta ^2} +4\frac{(\partial \psi )^2+ (\partial \chi )^2}{\eta }\;d^3x\nonumber \\&\, +\frac{1}{8\pi }\int _{\partial \varOmega }\partial _s\ln \rho \left( \ln \eta -\ln \rho \right) \;d^3x , \end{aligned}$$

where $\partial _s$ denotes the derivative in the (outward) direction normal to $\partial \varOmega$.

Here is where the connection with harmonic maps becomes evident. Recall that given the harmonic maps $(\eta ,\omega ,\chi , \psi ){:}\,\varOmega \subset {\mathbb {R}}^3{\setminus } \varGamma \rightarrow \mathbb {H}^2_{\mathbb {C}}$, where $\varGamma$ is the symmetry axis and $\mathbb {H}^2_{\mathbb {C}}$ is the hyperbolic complex plane, the energy $\tilde{\mathcal {M}}$ of such harmonic maps is defined by

165 $$\begin{aligned} \tilde{\mathcal {M}}_\varOmega :=\frac{1}{32\pi }\int _\varOmega \frac{(\partial \eta )^2}{\eta ^2}+ 4\frac{(\partial \omega +\chi \partial \psi -\psi \partial \chi )^2}{\eta ^2} +4\frac{(\partial \psi )^2+ (\partial \chi )^2}{\eta }\;d^3x.\nonumber \\ \end{aligned}$$

Therefore we have the relation

166 $$\begin{aligned} \mathcal {M}_\varOmega =\tilde{\mathcal {M}}_\varOmega +B_{\partial \varOmega } , \end{aligned}$$

where $B_{\partial \varOmega }$ is the boundary term introduced in (164).

We can apply the results of Hildebrandt et al. (1977) (see also Chruściel 2008) stating that when $\varOmega$ is compact and does not contain the axis $\varGamma$, and the target manifold has negative curvature (as $\mathbb {H}^2_{\mathbb {C}}$ in our case), the minimizers of $\tilde{\mathcal {M}}_\varOmega$ with Dirichlet boundary conditions exist, are unique, smooth and satisfy the Euler Lagrange equations.

Since the difference between $\mathcal {M}_\varOmega$ and the harmonic energy $\tilde{\mathcal {M}}_\varOmega$ is the boundary term $B_{\partial \varOmega }$, the minimizer of $\tilde{\mathcal {M}}_\varOmega$ is the minimizer of $\mathcal {M}_\varOmega$ as well. This minimizer is the extreme Kerr–Newman solution, and we obtain $\mathcal {M}_\varOmega \ge \mathcal {M}_\varOmega |_{\text{ ext } \text{ Kerr--Newman }}$.

After a subtle limit procedure that allows to extend the inequality valid in $\varOmega$ to all ${\mathbb {R}}^3$, one arrives at the desired inequality $\mathcal {M}\ge \mathcal {M}|_{\text{ extr } \text{ Kerr--Newman }}$.

### Cosmological constant

As we discuss in Sect. 3.2, Cha and Khuri (2017) study asymptotically AdS hyperbolic initial data and prove a global inequality where the cosmological constant does not appear explicitly. They conjecture, though, that an inequality of the form

167 $$\begin{aligned} M\ge & {} \frac{1}{3\sqrt{6}}\left[ \sqrt{\left( 1+\frac{J^2}{M^2}\right) ^2+\frac{12 J^2}{M^2}}+2\left( 1+\frac{J^2}{M^2}\right) \right] \end{aligned}$$

168 $$\begin{aligned}&\times \left[ \sqrt{\left( 1+\frac{J^2}{M^2}\right) ^2+\frac{12 J^2}{M^2}}-\left( 1+\frac{J^2}{M^2}\right) \right] ^{1/2} \end{aligned}$$

should hold, with equality in the extreme Kerr–Newman AdS black hole.

Related inequalities are given by Chruściel et al. (2006a), which is an extension of a previous work by Maerten (2006).

## Quasilocal inequalities for black holes

The geometrical inequalities presented in this section relate purely quasilocal quantities defined on a closed 2-surface. By this we mean that no *bulk* quantities defined on the 3-dimension region inside the surface are considered.

As is the case with global inequalities, here we divide the subject in three parts, the pure charge case, in Sect. 4.1, the inequalities involving angular momentum, in Sect. 4.2 and the inequalities involving (explicitly) a cosmological constant in Sect. 4.4. We do this for two reasons. First, the pure charge case problem does not need axial symmetry to be formulated, whereas it is needed in order to have a well defined quasilocal angular momentum (see Sect. 2). Also, even when the electric charge gives a flavor and a hint of what may happen with angular momentum, the techniques employed in the three treatments are usually different.

Let us analyze the settings and the factors that produce these quasilocal inequalities.

*Settings* As opposed to the global inequalities, which can be proven for complete initial surfaces, quasilocal inequalities need a well identified surface representing the black hole, where things are computed. Even then, the problem can be formulated from two different perspectives: a Riemannian and a Lorentzian points of view. These two approaches have to do with the different surfaces used in the derivation of the inequality. In the Riemannian setting, a minimal surface in an initial data set is studied. In the Lorentzian case, it is a MOTS in spacetime

*Inequality producer* In the quasilocal inequalities some form of a stability condition for the 2-surface considered is the main factor that produces the estimate. The Riemannian treatment requires the positivity of the second variation of the area function. In the Lorentzian setting the inequalities arise from stability of the MOTS.

### Area–charge

The relation between the area and the electromagnetic charges of a closed 2-surface was the first result obtained in the form of a quasilocal geometrical inequality.

It was first studied in a spacetime setting where trapping properties of 2-surfaces embedded in a Cauchy surface were assumed. Later, the inequality was proved purely from an initial data view point. And finally, from a purely Lorentzian one. We state a precise version of the result in the latter form below, and then discuss the different contributions, the hypotheses and conclusions.

#### Results

We extract the following theorem from the work of Dain et al. (2012).

##### Theorem 4

Given an orientable closed marginally trapped surface $\mathcal {S}$ satisfying the spacetime stably outermost condition, in a spacetime which satisfies Einstein equations, with non-negative cosmological constant $\varLambda$ and such that the non-electromagnetic matter fields satisfy the dominant energy condition, then the inequality

169 $$\begin{aligned} A\ge 4\pi Q^2 \end{aligned}$$

holds, where *A* is the area of $\mathcal {S}$ and $Q^2=Q_E^2 +Q_M^2$ is the total charge of $\mathcal {S}$.

We define the stability condition and address the other hypotheses below. Let us first review the different previous results on the subject.

Gannon (1976) obtains an inequality in the spirit of (169) when analyzing properties of electrovacuum black hole spacetimes that are strongly future asymptotically predictable from a partial Cauchy surface $\varSigma$ regular near infinity. This means that $\varSigma =\cup _{i=1}^\infty \varOmega _i$, with $\varOmega _i\subset \varOmega _{i+1}$ such that $\partial \varOmega _i:=\mathcal {S}_i$ are future inner trapped surfaces (i.e. $\theta _-<0$). These 2-surfaces are expected to be found in most isolated gravitating systems after moving sufficiently far from the center of the system and are the alternative to stable MOTS and minimal surfaces used by later authors. In this setting, Gannon proves that if the boundary of the black hole can be foliated by spacelike two-surfaces whose surface area is bounded above by $A_{\max }$, then $A_{\max }\ge 4\pi Q^2$, provided the electric charge *Q* is not zero (this restriction guarantees that the 2-surfaces are topological spheres). Strictly speaking, Gannon’s result is not quasilocal as it makes assumptions about all $\varSigma$. Nevertheless, we present the result here because of the great role it plays in the subject as a model and inspiration to subsequent work.

Later, Gibbons (1999), using the positivity of the second variation of the area function, obtains the inequality (169) as a particular case of results in higher dimensions. For our purposes here, he considers maximal, electrovacuum initial data with a stable minimal surface $\mathcal {S}$. Recall that given a maximal initial data $(\varSigma , h_{ij}, K_{ij}, \mu , j^i)$, and an embedded surface $\mathcal {S}$ in $\varSigma$, we say it is a minimal surface if the mean curvature of $\mathcal {S}$ vanishes and it is stable if the second variation of the area function is non-negative, namely $\delta ^2_{\alpha s}A\ge 0$ for all functions $\alpha$, where $s^i$ is a normal vector to $\mathcal {S}$ in $\varSigma$ (see Gibbons 1999, also Andersson et al. 2008a; Mars 2014). The stability condition gives the desired inequality (169) between the area *A* and the charge *Q* of $\mathcal {S}$. This result is a refinement of a previous work of Gibbons et al. (1996) in the spherically symmetric case.

In the Lorentzian settings, Dain et al. (2012) study the inequality between area and charge of a MOTS in a spacetime, satisfying certain stability property. Let us see it in some detail. Andersson et al. (2005, 2008b) introduce a notion of stability for a closed marginally trapped surface $\mathcal {S}$ which motivates the notion used in Theorem 4. $\mathcal {S}$ is said to be spacetime stably outermost if there exists an outgoing vector $X^\mu =f_\ell \ell ^\mu -f_k k^\mu$ with functions $f_\ell \ge 0$ and $f_k>0$ such that the variation $\delta _{X}$ of $\theta _+$ with respect to $X^\mu$ satisfies $\delta _{X}\theta _+\ge 0$, where $\delta$ is the variation operator associated with a deformation of $\mathcal {S}$. Charged matter fields are included, although the non-electromagnetic matter fields must satisfy the dominant energy condition. Extensions of this inequality are also proven for regions in the spacetime which are not necessarily black hole boundaries, but ordinary objects (see Sect. 5 for more details). They prove Theorem 4. They also prove a similar inequality to (169) for an oriented surface screening an asymptotically flat end. A screening surface of an end is a closed 2-surface that encloses an open, connected region $\varOmega \subset \varSigma$ which contains the mentioned end and no other.

As noted by Jaramillo (2013), the area charge inequality relies on the algebraic properties of the electromagnetic energy momentum tensor. This observation provides a straightforward generalization of (169) to other matter fields having similar algebraic properties. In particular, in Jaramillo (2013), the inequality (169) is extended to include the Yang–Mills charges.

#### Discussion

We wish now to make a few observations about this result.

As in the global case for the inequality between mass and charge (see the beginning of Sect. 3), no axial symmetry is required to obtain (169) due to the area and charge being well defined quasilocal quantities.

On the other hand, as opposed to the global inequalities involving the ADM mass, (169) is a quasilocal relation, it refers to the properties of one 2-surface describing the horizon. This means that if one considers a spacetime containing many black holes, that inequality should hold for each one of them. The paradigmatic example of this case is the Majumdar–Papapetrou solution (Majumdar 1947; Papapetrou 1945), which consists of an arbitrary number of extreme Reissner–Nordström-like black holes, all with charges of the same sign. This is a very special solution as each black hole saturates the bound (169), i.e., $A_i=4\pi Q_i^2$, where $A_i$ and $Q_i$ are the individual areas and charges respectively of each black hole.

The Majumdar–Papapetrou solution is static, nevertheless, inequality (169) holds in completely dynamical scenarios as well, even in the presence of charged matter fields satisfying the dominant energy condition. This is in contrast with the mass–charge inequality presented in Sect. 3.1, where a strong (and in a sense, unnatural) local condition is imposed on matter fields [see the discussion after Eq. (145)].

Note that there is no rigidity statement saying that if equality is attained in (169), then the solution must be the near horizon geometry of Reissner–Nordström.

In fact, the key ingredient used to prove Theorem 4 is the notion of stability for the 2-surface. This condition plays an analogous role as the non-negativity condition on the second variation of area function in Riemannian settings.

### Area–angular momentum–charge

At the beginnings of 2007, two results relating horizon area and angular momentum of black holes were given, one for stationary black holes by Ansorg and Pfister and the other for isolated horizons by Booth and Fairhurst (see the *Living Review* by Ashtekar and Krishnan 2004 for definitions and general results on isolated horizons). These two works have motivated the more general study of quasilocal inequalities that explicitly include the angular momentum of a given surface in a dynamical system.

The different settings where the desired inequality have been proven are stationary spacetimes, maximal initial data and finally trapped surfaces.

#### Results

We present here one of the results, taken from the article by Gabach Clément et al. (2013) that is valid for dynamical as well as stationary black holes represented by an appropriate closed 2-surface.

##### Theorem 5

Let $\mathcal {S}$ be either

1. a smooth spacetime stably outermost axisymmetric marginally outer trapped surface (MOTS) embedded in a spacetime, satisfying the dominant energy condition, or
2. a smooth stable axisymmetric minimal surface in a maximal data set, with non-negative scalar curvature,

with a non-negative cosmological constant $\varLambda$, angular momentum *J*, charges $Q_E$ and $Q_B$ and area *A*. Then,

170 $$\begin{aligned} A\ge \sqrt{(8\pi J)^2+(4\pi Q^2)^2} \end{aligned}$$

with $$Q^2=Q_E^2+Q_B^2$$.

Moreover, the equality in (170) is achieved if and only if the surface is the extreme Kerr–Newman sphere.

We review the many results that led to this theorem and discuss the theorem afterwards, in Sect. 4.2.2.

The first, known to us, work on a geometrical inequality in the spirit of (170) is due to Ansorg and Pfister (2008). They treat stationary and degenerate black holes and find that they must satisfy the equality in (170). More precisely, the equality in (170) holds for every element in a parametric sequence of axially and equatorially symmetric, stationary systems consisting of a degenerate black hole surrounded by matter such that the limit system is the Kerr–Newman black hole. Moreover, the authors conjecture that for axially and equatorially symmetric, stationary black holes surrounded by matter, the inequality (170) should hold, with equality at the degenerate case.

A few months later, Booth and Fairhurst (2008) argue that the allowed values for the angular momentum of an isolated horizon $\mathcal {S}$ should be determined from the intrinsic horizon geometry. They find

171 $$\begin{aligned} 2\sqrt{e\gamma }A\ge 8\pi |J| \end{aligned}$$

where *e* is the surface integral of the evolution equation for the inward expansion at the horizon and $\gamma :=\pi A^{-2}\int _S\eta$, where $\eta =\eta ^i\eta _i$ is the square norm of the rotation vector field. Moreover, $e\le 1$ and $e=1$ at an extremal horizon and $\gamma <1/4$ for axially symmetric horizons whose cross sections can be embedded in Euclidean space. In that case, i.e., when $\gamma <1/4$, one obtains the strict inequality

172 $$\begin{aligned} A>8\pi |J|. \end{aligned}$$

However, the authors argue that otherwise, $\gamma$ can become arbitrarily large making the bound (171) to lose its meaning.

The Ansorg and Pfister conjecture is finally proven in vacuum by Hennig et al. (2008, 2010), and Ansorg et al. (2011). They show that every axially symmetric and stationary black hole with surrounding matter satisfies (170) and equality holds if the black hole is extremal. The proof consists in showing that if $A\le \sqrt{(8\pi J)^2+(4\pi Q^2)^2}$, then the black hole can not be subextremal in the sense of Booth and Fairhurst (2008). Recall that a black hole is subextremal if there exist trapped surfaces in every small interior vicinity of the event horizon. Einstein equations near the horizon are then considered and a variational problem is formulated and solved. As we discuss in Sect. 4.2.2 this stationary variational problem is closely related to the variational problem arising in the dynamical regime.

Dain (2010) conjectures the validity of the vacuum case of (170) for the connected component of the apparent horizon in a dynamical scenario. The proof was given later, as in the pure charge case, from two perspectives, one Riemannian and one Lorentzian.

With a Riemannian approach, Aceña et al. (2011) prove that extreme Kerr initial data is the global minimum for certain mass functional $\mathbb {M}$ related to the second variation of the area functional and analogous to the mass functional $\mathcal {M}$ presented in Sect. 3.2.5. They prove the inequality (170) with $Q=0$ for vacuum axially symmetric initial data containing a minimal surface and such that the metric $h_{ij}$ satisfies certain technical conditions. However, this class considered includes many known black hole initial data. They extend the validity of the inequality to include a non-negative cosmological constant, not appearing explicitly into the inequality though. However, this generalization is relevant because there exists a counter-example of the inequality (170) with $Q=0$ for the case of negative cosmological constant, as it was pointed out in Booth and Fairhurst (2008).

In Gabach Clément (2011), the author relaxes some of the restriction on the type of surfaces studied and Dain and Reiris (2011) prove the inequality (170) with $Q=0$, in vacuum, replacing the technical conditions of Aceña et al. (2011) by the stability condition on the minimal surface (see Sect. 4.1.1 for definition of stable minimal surface).

With a Lorentzian treatment, Jaramillo et al. (2011) prove (170) for an axially symmetric closed marginally trapped surface $\mathcal {S}$ satisfying the spacetime stably outermost condition (see Sect. 4.1.1), in a spacetime with non-negative cosmological constant and matter fields satisfying the dominant energy condition. The rigidity statement with the extreme Kerr sphere, instead of the extreme Kerr–Newman sphere is also proven for the $Q=0$ case.

The inclusion of electromagnetic charges is done by Gabach Clément and Jaramillo (2012) and by Gabach Clément et al. (2013). Through the introduction of appropriate electromagnetic and angular momentum potentials, they prove Theorem 5 in two different ways and show the connection with the variational problem for the stationary case (see the remarks below). Also a relation between the two mass functionals $\mathcal {M}$ introduced in Sect. 3.2.5, Eq. (160) and $\mathbb {M}$ is pointed, which suggests a relation between the global inequality (146) and the quasilocal inequality (170). We explore this in Sect. 4.2.5 with more detail.

In Gabach Clément (2012), an extension of (170) to systems of many black holes with struts are obtained, and in Manko et al. (2013, 2014), Cabrera-Munguia et al. (2013) and Cabrera-Munguia (2015), binary systems saturating this inequality are presented.

#### Discussion

We wish to make a few remarks about the hypotheses and statements of Theorem 5.

Theorem 5 calls for stable closed surfaces. As we mention at the beginning of Sect. 4, it is this stability property what drives the inequalities. Both concepts of stability appeared already in problem with no angular momentum, see Sect. 4.1 for definitions. The only extra assumptions we make in this result is that the functions $\alpha$ and $f_\ell , f_k$ entering the stability criteria for minimal surfaces and MOTSs respectively must be axially symmetric. In Sect. 4.2.5 the connection between these two stability conditions is revised. The interesting point made in Gabach Clément et al. (2013) is that the corresponding stability assumptions both for minimal surfaces and MOTS lead to exactly the same integral condition.

The theorem admits a non-negative cosmological constant, but it does not enter explicitly into the inequality. The treatment of such problem needs different techniques and is reviewed in Sect. 4.4. The relevant property of the cosmological constant in this theorem is that its positivity allows one to disregard it altogether from the Einstein or constraint equations. Clearly, the same can not be made for negative $\varLambda$ and the problem of determining and proving the appropriate inequality for negative $\varLambda$ is still open (see Sect. 4.4).

Non-electromagnetic matter fields are admitted in the hypotheses of Theorem 5 and they are not required to satisfy the dominant energy condition. Only the complete energy momentum tensor $T_{\mu \nu }^M+T_{\mu \nu }^{{\textit{EM}}}$ must satisfy the dominant energy condition. Also there can be matter surrounding and crossing the surface $\mathcal {S}$. This in particular extends the results in Ansorg et al. (2011) and Hennig et al. (2010).

A major difference between Theorems 4 and 5 is the rigidity statement. The horizon in extreme Reissner–Nordström clearly saturates inequality (169), but so far it is not proven that it is the only horizon that does. On the other hand, the horizon in extreme Kerr–Newman is the unique solution that satisfies the equality in (170). In the latter case, it is the connection between a certain mass functional $\mathbb {M}$ and the energy of harmonic maps and the uniqueness of minimizers of that energy what ultimately gives uniqueness in Theorem 5. See Sect. 4.2.5 for details.

The extreme Kerr–Newman sphere mentioned in Theorem 5 has a precise meaning in terms of intrinsic and extrinsic quantities defined on the surface $\mathcal {S}$ (Gabach Clément et al. 2013). Basically the surface has the geometry of a horizon section in the extremal Kerr–Newman black hole. In Sect. 4.2.5 we give proper definitions, here, however, we want to emphasize that the rigidity statement refers to the extreme Kerr–Newman horizon, not the entire initial data. Interestingly, the fact that the equality in (170) is only attained by the extreme Kerr–Newman sphere has been known since the work of Hájiček (1974), Lewandowski and Pawlowski (2003), and Kunduri and Lucietti (2009, 2013) on isolated horizons and near horizon geometries of extreme black holes. See also the more recent results of Reiris (2014a) and Chruściel et al. (2017).

Finally, we want to mention the relation between the variational problem used to prove Theorem 5 and the one used in the stationary case in Ansorg et al. (2011). The argument in Ansorg et al. (2011) to prove the strict inequality (170) is based on the implication

173 $$\begin{aligned} \text{ subextremal } \text{ horizon }\quad \Rightarrow \quad A>\sqrt{(8\pi J)^2+(4\pi Q^2)^2}. \end{aligned}$$

The counterreciprocal of (173) is written as a variational problem for an action functional on a Killing horizon section. As it is shown in Gabach Clément and Jaramillo (2012), this action and variational problem are identical to the corresponding mass functional $\mathbb {M}$ and the variational problem formulated for a stable MOTS. This connection is particularly remarkable. We mentioned already a link between the variational problems for stable minimal surfaces and stable MOTS. This is essentially a manifestation of the close relation between the two different characterizations of black holes. However, the great similarities with the stationary case are not at all a priori obvious, especially considering that the treatment in Ansorg et al. (2011) makes use of the particular form of the 4-dimensional stationary, axially symmetric metric, whereas the arguments in the proof of Theorem 5 refer solely to the stable surface $\mathcal {S}$.

#### Area products

We want to mention a close relation valid for axially symmetric, charged, rotating and stationary black holes with surrounding matter. It not only involves the event horizon area, *A*, but also the Cauchy horizon area $A_{\text{ Cauchy }}$. It reads

174 $$\begin{aligned} (8\pi )^2\left( J^2+\frac{Q^4}{4}\right) =AA_{\text{ Cauchy }} \end{aligned}$$

The remarkable observation is that the area product does not depend on the total mass, Eq. (174) is quasilocal. Is is a consequence of the fact that there can not be matter between the event and Cauchy horizon due to stationarity. Equation (174) has been proven by Ansorg and Hennig (2008, 2009), Hennig and Ansorg (2009) and by Ansorg et al. (2011). See also Visser (2013) on the validity of such mass independent, area-related functions. It also received a huge interest in string and other theories, see the work by Cvetic et al. (2011a) and the review by Compere (2017) on the Kerr/CFT correspondence.

#### Shape of black holes

A closely related quasilocal inequality for black holes is obtained by Gabach Clément and Reiris (2013). They link black-hole shape parameters with angular momentum. More precisely, for a rotating, axially symmetric spacetime stably outermost horizon, the length ${\mathcal {C}}_e$ of the greatest axially symmetric circle and the length of the meridian *L* satisfy

175 $$\begin{aligned} \frac{16\pi ^2|J|}{\sqrt{4\pi A}}\le & {} {\mathcal {C}}_e\le \sqrt{4\pi A} \end{aligned}$$

176 $$\begin{aligned} 4|J|\le & {} \frac{A}{2\pi }\le L^2 \end{aligned}$$

and

177 $$\begin{aligned} \frac{A}{L^2}\le \frac{{\mathcal {C}}_e}{L}\le 2\sqrt{2}\pi . \end{aligned}$$

There are three effects that show up in these results. The most expected one is a thickening of the bulk of the horizon due to rotation. They also show that rotation stabilizes the horizon’s shape in that the area and angular momentum control completely its local shape. Finally, at high angular momentum, the geometry of the horizon goes to that of extreme Kerr horizon, even in non-vacuum.

#### The mass functional $\mathbb {M}$

Analogous to the global case, the proof of the quasilocal inequality (170) is based on some remarkable properties of a quasilocal mass functional $\mathbb {M}$ and consists of two intermediate inequalities for $\mathbb {M}$. Again, for simplicity, we assume electrovacuum.

The proof consists of two steps. The first one is to prove a lower bound on the area of the 2-surface $\mathcal {S}$ in terms of a mass functional $\mathbb {M}$. One starts with the appropriate stability condition (for either type of surface, minimal or marginally outer trapped) and proves $A\ge 4\pi e^{\frac{\mathbb {M}-8}{8}}$. The second step is to prove that extreme Kerr–Newman horizon is the unique minimizer for $\mathbb {M}$, that is $\mathbb {M}\ge \mathbb {M}|_{\text{ extr } \text{ Kerr--Newman }}$. We follow Gabach Clément et al. (2013).

**Step 1.** $$A\ge 4\pi e^{\frac{\mathbb {M}-8}{8}}$$

For an axially symmetric, stable, minimal surface $\mathcal {S}$, the stability condition $\delta ^2_{\alpha s}A\ge 0$ is written in an integral form as

178 $$\begin{aligned} \int _{\mathcal {S}}|D\alpha |^2+\frac{{}^2R}{2}\alpha ^2 \; ds\ge \int _{\mathcal {S}}\frac{1}{2}\left( {}^3R+|\varTheta |^2\right) \alpha ^2\; ds\end{aligned}$$

for arbitrary and axially symmetric functions $\alpha$, and where ${}^2R$ and ${}^3R$ are the scalar curvature of the metrics on $\mathcal {S}$ and on $\varSigma$ respectively. $\varTheta$ is the traceless part of the extrinsic curvature of $\mathcal {S}$. The norms and surface element $ds$ are computed with respect to the intrinsic metric on $\mathcal {S}$.

On the other hand, for an axially symmetric, spacetime stably outermost MOTS $\mathcal {S}$, the stability condition $\delta _{X}\theta _+\ge 0$ with $X^\mu =\alpha \ell ^\mu +\varPsi k^\mu$ where $\alpha >0$ and $\varPhi \ge 0$ are axially symmetric arbitrary functions, can be written (after using Einstein equations and disregarding terms with the appropriate sign)

179 $$\begin{aligned} \int _{\mathcal {S}}|D\alpha |^2+\frac{{}^2R}{2}\alpha ^2 \; ds\ge \int _{\mathcal {S}}|\varUpsilon ^{(\eta )}|^2+E_\perp ^2+B_\perp ^2\; ds, \end{aligned}$$

where $\varUpsilon ^{(\eta )}$ is the projection on the axial Killing vector $\eta$ of the normal fundamental form of $\mathcal {S}$ and $E_\perp :=\ell ^\mu k^\nu F_{\mu \nu }$, $B_\perp :=\ell ^\mu k^\nu {}^*F_{\mu \nu }$ are the electromagnetic fluxes across the surface $\mathcal {S}$.

The two inequalities (178) and (179) become identical when the Hamiltonian constraint (149) is inserted in (178) and the relation $\varUpsilon ^{(\eta )}_\mu \eta ^\mu =-K_{\mu \nu }\eta ^\mu s^\nu$ is considered. Here $s^\mu$ is a spacelike normal to $\mathcal {S}$.

One starts by writing the metric on the surface as (see Dain and Reiris 2011 where such coordinate system is constructed)

180 $$\begin{aligned} ds^2=e^{2c-\sigma }d\theta ^2 +e^{\sigma }\sin ^2\theta d\varphi ^2 \end{aligned}$$

where $\sigma =\sigma (\theta )$ and *c* is a constant related to the area of $\mathcal {S}$ by $A=4\pi e^c$ and to $\sigma$ by

181 $$\begin{aligned} \sigma |_{\theta =0,\pi }=c . \end{aligned}$$

In these coordinates, the axial Killing vector field on $\mathcal {S}$ is $\eta ^i=\partial _\varphi ^i$ and its square norm is given by $\eta =e^\sigma \sin ^2\theta$. The component $\varUpsilon ^{(\eta )}$ of the normal form can be written in terms of a function $\tilde{\omega }$ as

182 $$\begin{aligned} \varUpsilon ^{(\eta )}_\theta =0,\quad \varUpsilon ^{(\eta )}_\varphi =-e^{\sigma -c}\sin \theta \tilde{\omega }' . \end{aligned}$$

where a prime denotes derivative with respect to $\theta$.

As in the proof of the global inequality, suitable potentials $\psi , \chi , \omega$ for the electromagnetic fields and rotation are introduced via the equations

183 $$\begin{aligned} \psi '= & {} -E_\perp e^c\sin \theta ,\quad \chi '=-B_\perp e^c\sin \theta \end{aligned}$$

184 $$\begin{aligned} \omega '= & {} 2\eta \tilde{\omega }'-2\chi \psi '+2\psi \chi '. \end{aligned}$$

Note that we use the same letters to denote these 2-dimensional potentials and the 3-dimensional potentials introduced in section Sect. 3.2.5. As the latter, the potentials defined by (183), (184) have the important property

185 $$\begin{aligned} Q_E=\frac{\psi (\pi )-\psi (0)}{2},\quad Q_B=\frac{\chi (\pi )-\chi (0)}{2},\quad J=\frac{\omega (\phi )-\omega (0)}{8}, \end{aligned}$$

where the charges and angular momentum refer to the surface $\mathcal {S}.$

Writing the stability condition in terms of these potentials, and setting the arbitrary function $\alpha$ to be

186 $$\begin{aligned} \alpha =e^{c-\sigma /2} \end{aligned}$$

(see Gabach Clément et al. 2013 for a discussion about this choice) one finds

187 $$\begin{aligned} \frac{A}{4\pi }\ge e^{\frac{\mathbb {M}-8}{8}} , \end{aligned}$$

where the mass functional $\mathbb {M}$ is defined by

188 $$\begin{aligned} \mathbb {M}:=\frac{1}{2\pi }\int _{S^2}\left[ 4\sigma +(\sigma ')^2+\frac{(\omega '+2\chi \psi '-2\psi '\chi ')^2}{\eta ^2}+ 4\frac{(\psi ')^2+(\chi ')^2}{\eta }\right] ds_0 , \end{aligned}$$

where the norms and surface element are computed with respect to the round metric on the unit sphere $d\theta ^2+\sin ^2\theta d\varphi ^2$. The great resemblance between $\mathbb {M}$ and the mass functional $\mathcal {M}$ defined in (160) is discussed in Sect. 4.3.

One of the most remarkable property of the functional $\mathbb {M}$ is that the boundary conditions for the functions it depends on are the angular momentum and charges (185) and the area (181). This is especially relevant for the formulation and solution of the variational problem in the next step.

**Step 2.** $$\mathbb {M}\ge \mathbb {M}|_{\text{ extr } \text{ Kerr } \text{ Newman }}$$

The second step is the resolution of a variational principle for the functional $\mathbb {M}$ giving the global minimum in terms of the angular momentum and charges:

189 $$\begin{aligned} e^{\frac{{\mathbb {M}}-8}{4}}\ge \ 4J^{2} + Q^{4} , \end{aligned}$$

with $$Q^2=Q_{E}^2 + Q_{B}^2$$.

The proof of the inequality (189) can be approached in several ways, as presented in Gabach Clément and Jaramillo (2012) and Gabach Clément et al. (2013). We already commented the reduction to the variational problem in stationary settings. Here we mention the other two arguments.

One of them follows the lines of Step 2 in Sect. 3.2.5. Here, the energy $\tilde{\mathbb {M}}$ of harmonic maps $(\eta ,\omega ,\chi ,\psi ){:}\,U\subset S^2{\setminus }\{\theta =0,\pi \}\rightarrow \mathbb {H}^2_{\mathbb {C}}$ is considered. It reads

190 $$\begin{aligned} \tilde{\mathbb {M}}_U:=\frac{1}{2\pi }\int _U\left[ \frac{(\eta ')^2}{\eta ^2}+\frac{(\omega '+2\chi \psi '-2\psi '\chi ')^2}{\eta ^2}+ 4\frac{(\psi ')^2+(\chi ')^2}{\eta }\right] ds_0 . \end{aligned}$$

There are two differences between (188) and (190). One is the integration region, $\mathbb {M}$ involves an integration over $S^2$ while $\tilde{\mathbb {M}}$ involves the integration over $U\subset S^2$. The second difference concerns the integrand. $\mathbb {M}$ contains the integral of $\sigma$ and $\sigma '$, while $\tilde{\mathbb {M}}$ contains the integral of $\eta$ and $\eta '$. when we restrict the integral in the definition of $\mathbb {M}$ to the region *U* and denote it by $\mathbb {M}_U$, we obtain the relation

191 $$\begin{aligned} \tilde{\mathbb {M}}_U=\mathbb {M}_U+4\int _U\ln \sin \theta \, ds+\oint _{\partial U}(4\sigma +\ln \sin \theta )\partial _\nu \ln \sin \theta \, dl \end{aligned}$$

where $\partial _\nu$ is the derivative in the direction of the exterior unit vector normal $\nu$ to $\partial U$ and *dl* is the line element in $\partial U$. We see that the difference between $\mathbb {M}_U$ and $\tilde{\mathbb {M}}_U$ is a constant plus a boundary term, which implies that both functionals have the same Euler–Lagrange equations. The result of Hildebrandt et al. (1977) is again used to give existence of a unique minimizer for $\tilde{\mathbb {M}}_U$. That minimizer is the extreme Kerr–Newman sphere, defined as the set $(\sigma _0,\omega _0, \chi _0,\psi _0)$ that can be obtained by computing the geometry on a horizon section of the extreme Kerr–Newman solution.

Finally a very subtle limit procedure must be performed to arrive at the desired inequality $\mathbb {M}\ge \mathbb {M}|_{\text{ extr } \text{ Kerr } \text{ Newman }}$.

It is worth mentioning that the previous variational problem can be solved without assuming axial symmetry on the functions $\sigma ,\omega , \psi ,\chi$.

The second approach is restricted to axial symmetry and hence in the minimization problem for $\mathbb {M}$, the Euler–Lagrange equations reduce to a system of ordinary differential equations. When solving these equations, the boundary conditions *J*, $Q_{E}$ and $Q_{ M}$ determine uniquely the boundary conditions for the remaining potential $\sigma$. This is the key fact under the sharpness of inequality (170). A constructive explicit proof of existence and uniqueness for the minimizer of $\mathbb {M}$ is given in Gabach Clément et al. (2013) with prescribed values of $J, Q_{ E}, Q_{ M}$ and without any reference to the boundary values of $\sigma$. This is different to what one does in the first approach discussed above, where the boundary values of $\sigma$ are prescribed from the relation $A=4\pi e^{\sigma }|_{\theta =0}$ valid for the particular coordinate system employed.

### Relation between $\mathcal {M}$ and $\pmb {\mathbb {M}}$

It is remarkable that both, the global (146) and the quasilocal (170) inequalities involving angular momentum are derived from mass functionals $\mathcal {M}$ and $\mathbb {M}$ respectively, and that these functionals are minimized by some form of the extreme Kerr–Newman solution. In Gabach Clément et al. (2013), a connection between these two functionals is presented, which in turn, gives a connection between the two inequalities.

More precisely, it is shown there that the inequality $m\ge \mathcal {M}\ge \mathcal {M}_0$ implies that the extreme Kerr–Newman horizon is a local minimum of the mass functional $\mathbb {M}$, which suggests that the global inequality (146) implies the quasilocal inequality (170):

192 $$\begin{aligned} M^2\ge \frac{Q_\infty ^2+\sqrt{Q_\infty ^4+4J_\infty ^2}}{2} \quad \Rightarrow \quad A\ge \sqrt{(8\pi J)^2+(4\pi Q^2)^2} . \end{aligned}$$

On the other hand, Penrose inequality together with the quasilocal inequality (170) give

193 $$\begin{aligned} \left[ M^2\ge \frac{A}{16\pi }\right] \quad +\quad \left[ A\ge \sqrt{(8\pi J)^2+(4\pi Q^2)^2}\right] \quad \Rightarrow \quad M^2\ge \frac{A}{16\pi }\ge \frac{4J^2+Q^4}{4} \end{aligned}$$

for stable MOTS, which is a weaker version of the global inequality (146).

Whether there exist a deeper connection and a full implication of the form

194 $$\begin{aligned} M^2\ge \frac{Q^2+\sqrt{Q^2+4J^2}}{2} \quad \Longleftrightarrow \quad A^2\ge (8\pi J)^2+(4\pi Q^2)^2 \end{aligned}$$

is far from settled. See also the discussion given in Sect. 1.1 about this issue in the context of stationary black holes.

### Cosmological constant

The results of Sects. 4.1 and 4.2 admit non-negative cosmological constants, but do not include them explicitly into the inequalities. These results are presented in this section as, in general, they require different techniques.

By analyzing explicit solutions and collapsing black holes, Hayward et al. (1994) and Shiromizu et al. (1993), prove that a positive cosmological constant sets restrictions on how large a black hole can be (see also Maeda et al. 1998). They study black-hole spacetimes with positive cosmological constant $\varLambda$, that satisfy the dominant energy condition, and find that the area of the black hole horizon, as described by an outer marginal surface, is bounded as $A\le 4\pi /\varLambda$. The same inequality holds for the area of a connected section of the event horizon in the case of strongly future asymptotically predictable, asymptotically de Sitter spacetime. The inequality is saturated for the extreme Schwarzschild–de Sitter horizon.

For negative $\varLambda$, Gibbons (1999) (in the time symmetric settings) and Woolgar (1999) (in the non-time-symmetric case) find the bound $A>4\pi (g-1)/|\varLambda |$, for the area of an outermost MOTS of genus $g>1$.

These inequalities show the important role that the cosmological constant plays in determining the size of a black hole. Note in particular, that the positive and negative cosmological constants bound the area in opposite directions. Namely a de Sitter-like black hole can not be too large and an anti de Sitter-like black hole can not be too small. This has interesting implications for studying possible colliding scenarios.

Gibbons (1999) also considers the combined effect of a cosmological constant and matter fields satisfying the dominant energy condition. He finds that the area of a stable minimal surface $\mathcal {S}$ in a time symmetric 3-surface is bounded as $4\pi (1-g)-\varLambda A-\int _S8\pi T_{00}>0$, where $T_{00}$ is the energy density of the matter fields on the 3-surface.

Simon (2012) arrives at the same inequality for stable MOTS in a spacetime satisfying the dominant energy condition. Interestingly, when Maxwell fields are explicitly taken into account, he is able to write the inequalities as

195 $$\begin{aligned}&2\pi \left( 1-\sqrt{1-4\varLambda Q^2}\right) \le \varLambda A\le 2\pi \left( 1+\sqrt{1-4\varLambda Q^2}\right) \quad \varLambda >0, \end{aligned}$$

196 $$\begin{aligned}&2\pi \left[ g-1+\sqrt{(g-1)^2-4\varLambda Q^2}\right] \le -\varLambda A\quad \varLambda <0 . \end{aligned}$$

Note that Simon (2012) maintains the (non-negative) principal eigenvalue of the stability operator in his inequality. We omit it here for simplicity. Inequalities (195) show that for positive cosmological constant one obtains both an upper and a lower bound to the area. This in essence is a manifestation of a competition of two effects. On one hand, the charges forbid the black hole to become too small (due to electric repulsion). On the other hand, the positive cosmological, through the cosmological radius, sets an upper limit to the horizon area. Inequality (196) shows that when $\varLambda$ is negative, both effects, the ‘cosmological’ and the electric repulsion combine to give a lower bound to the area.

The inequalities (195)–(196) are saturated in spherical symmetry by the Reissner–Nordström–de Sitter black holes if and only if the surface gravity vanishes. Simon also discusses the time evolution of MOTSs and the application of his inequalities as restrictions on the merging. He deduces an interesting Corollary, non-trivial only when $\varLambda \ne 0$, which gives lower and upper bounds on the quotient between the *initial* and *final* areas of MOTSs (homologous MOTSs). This result is applied to the situation where only a single MOTS is *initially* present, and to the problem of merging of MOTSs.

As has been previously pointed out, the inclusion of angular momentum into geometrical inequalities requires different techniques. The extension of (195) to rotating black holes was done by Gabach Clément et al. (2015). They consider an axially symmetric, stable MOTS, with $\varLambda >0$ and matter satisfying the DEC, and find that the allowed values of angular momentum *J* are given by

197 $$\begin{aligned} |J|\le \frac{A}{8\pi }\sqrt{\left( 1-\frac{\varLambda A}{4\pi }\right) \left( 1-\frac{\varLambda A}{12\pi }\right) } , \end{aligned}$$

where *A* is the area of the MOTS. The inequality (197) is saturated by the extreme Kerr–deSitter horizons. One can read from (197) that the presence of a positive cosmological constant sets stronger limits to the allowed values of the angular momentum. Namely, a cosmological horizon must rotate more slowly than the non-cosmological one. This observation agrees with the intuitive idea mentioned above, that the cosmological constant has an attractive effect. Hence, if the area is fixed, then the rotation must be slowed down. Another way of looking at (197) is to consider the right-hand side of (197) as an effective area $A_{eff}$, with $A_{eff}\le A$ due to being $\varLambda >0$. In this notation, (197) reads $|J|\le A_{eff}/8\pi$. It is worth remarking that the proof of inequality (197) follows the lines of the proof the Area–Angular momentum inequality (see Sect. 4.2.5), in that the stability condition is used to obtain a lower bound on the area in terms of a mass functional $\mathbb {M}^\varLambda$ defined by

198 $$\begin{aligned}&\mathbb {M}^\varLambda (\sigma ,\omega , A,a)\nonumber \\&\quad :=\frac{1}{2\pi }\int \left[ \sigma '^2+\frac{\omega '^2}{\eta ^2}+4\sigma \frac{1+\varLambda a^2\cos ^2\theta }{\zeta }+ 4\left( \frac{A}{4\pi }\right) ^2\varLambda e^{-\sigma }\right] \zeta \; d^3x,\qquad \qquad \end{aligned}$$

where

199 $$\begin{aligned} \zeta :=1+\frac{\varLambda a^2\cos ^2\theta }{3} \end{aligned}$$

and we have explicitly written the elements that $\mathbb {M}^\varLambda$ depends on, because they make the variational principle much harder than when one bounds away the cosmological constant (as was shown in Sect. 4.2.5). The first difficulty that arises when one keeps the term containing $\varLambda$ (to ensure that it will come up in the final inequality) is that the mass functional also depends explicitly on *A*

The second difficulty is proving existence and uniqueness of a minimizer for $\mathbb {M}^\varLambda$, as there is no direct relation between $\mathbb {M}^\varLambda$ and energy of harmonic maps. These obstacles are overcome as follows. The first one is dealt with by a scaling argument where *A* and *J* are frozen to the extreme Kerr–deSitter values and the dynamical variables in $\mathbb {M}^\varLambda$ change appropriately. For the second one, it is proven that every critical point of $\mathbb {M}^\varLambda$ is a local minimum and then the mountain pass theorem is used to obtain the global existence. In the presence of Maxwell fields, the inequality

200 $$\begin{aligned} J^2\le \frac{A^2}{64\pi ^2}\left[ \left( 1-\frac{\varLambda A}{4\pi }\right) \left( 1-\frac{\varLambda A}{12\pi }\right) -\frac{2\varLambda Q^2}{3}\right] -\frac{Q^4}{4} \end{aligned}$$

is conjectured to hold in Gabach Clément et al. (2015) under the same hypotheses.

Inequality (200) was proven by Bryden and Khuri (2017) following the same ideas as in Gabach Clément et al. (2015), but simplifying the resolution of finding the minimizer of $\mathbb {M}^\varLambda$. The argument is based on the result of Schoen and Zhou (2013), which states that $\mathbb {M}^\varLambda$ is convex along geodesic deformations within $\mathbb {H}^2_{\mathbb {C}}$.

The case of negative cosmological constant is considerably more complicated as $\varLambda$ appears with the wrong sign in the mass functional $\mathcal {M}$. In a different context, Kunduri and Lucietti (2009, 2013) prove that the near horizon geometry of axisymmetric and stationary black holes is the one of the extremal Kerr–Newmann-anti-de Sitter horizon and therefore they saturate (200) (see Hennig 2014 for an explicit expression and discussion).

### An application: non-existence of two black holes in equilibrium

A very interesting application of the area–angular momentum inequality (170), with $Q=0$, is the result by Neugebauer and Hennig (2009, 2012, 2014) and Hennig and Neugebauer (2011) where they prove that a two black hole configuration in equilibrium does not exist. This problem has been open since the early days of General Relativity (see Neugebauer and Hennig 2014 for further references and Beig and Chruściel 1996; Beig and Schoen 2009; Manko et al. 2011 for different approaches and results on the subject); an alternative proof of non-existence was given by Manko and Ruiz (2001). The Neugebauer and Hennig argument is the following: Start out with the spacetime metric for an axially symmetric, stationary system containing two disconnected Killing horizons on the symmetry axis. Use the Ernst formulation Ernst (1968) to obtain a system of equations equivalent to Einstein vacuum equations. Then the inverse scattering method is used to build a unique and exact solution to the Ernst equations, known as the double Kerr-NUT solution. A particular property of this solution is that both black holes can not satisfy the $A\ge 8\pi |J|$ inequality simultaneously, which proves the non-existence of two black holes in equilibrium. This result was generalized by Chruściel et al. (2011) to $I^+$ regular black hole spacetimes.

## Inequalities for objects

The inequalities presented in Sects. 3 and 4 are valid for black holes. The presence of such black hole is manifested through the hypothesis of the existence of a trapped surface or of a non-trivial topology in the initial data.

The interest in geometrical inequalities for ordinary objects is twofold. The most basic question is whether Einstein equations set restrictions on the values that physical parameters for objects can attain. This is not the case in Newtonian theory unless some specific matter model with intrinsic restrictions is used. Is this the case in General Relativity? Are there some conditions on the mass, size, rotation, and charge of an object, such that if they are not fulfilled, the object can not exist within the theory? This is related to the second question we want to address in this section. Are there geometrical inequalities for objects such that if violated, the object collapses to form a black hole? Clearly, the formation of a black hole after the collapse of an ordinary object is one possible scenario leading to the non-existence raised in the first question. However, we emphasize that these two situations, i.e., *an object exists and satisfies certain inequalities*, and *an object does not exist because it forms a black hole* are in principle very different and require different treatments.

The problem of finding geometrical inequalities in the non-black hole setting is wide open. At this point it is not all that clear what kind of inequalities one should look for (some of them have been motivated in the introduction though, see Sect. 1.3), nor what the proper systems and physical quantities are, that will produce such inequalities (we discuss this point below). This makes research in this area look a bit erratic, where new ideas are proposed or applied in almost every article. Because of this, we choose to present the results and discussions in a different manner as we do in previous sections. We discuss the general problems first and then present the results with specific remarks for them.

### Discussion

There are two major differences between non-black hole objects and black holes. The first is the problem of how to characterize the object in such a way that it produces the desired relation between physical quantities (like mass, electromagnetic charges or angular momentum) and size or shape parameters. That is, we would need some positivity condition to play the role of the stability of MOTS or minimal surfaces used for obtaining black hole inequalities (a non-trivial topology for the underlying initial surface is not considered when studying physically reasonable ordinary objects).

The second problem, maybe less challenging but still open, is how to properly measure the object. When non-black hole objects are considered, one may want to consider measures of 3-dimensional subsets of an initial data, and not just measures of 2-surfaces. This raises several difficulties as there does not seem to exist consensus about what the best or more appropriate measure is for the size of a non-black hole object. Indeed, a proper and suitable measure of size of an object should satisfy certain requirements. Namely, it should give a good, intuitive idea of size, it should be relatively easy to compute, it should be so chosen as to actually appear in the aimed geometrical inequalities.

These problems are aggravated by the fact that in general, there is not a special non-trivial ordinary object known to saturate an estimate of the form

201 $$\begin{aligned} {[}\text{ Size }]\gtrsim \text{[Mass] } \text{ or } \text{[Angular } \text{ momentum] } \text{ or } \text{[Charge] }, \end{aligned}$$

where the symbol $[\cdot ]$ indicates only the dependence of each term (by applying dimensional arguments one could propose a great number of more precise inequalities). This leaves us without a model solution to look at, as opposed to the extreme black holes in black hole inequalities. In fact, if such paradigmatic fully relativistic object satisfying certain geometrical inequality existed (as extreme Kerr–Newman black hole in the black hole scenario), it would give us a path to what kind of inequality we should look for.

Note that to explore the rigidity case in (201) means to address the problem of minimizing [Size] for given [Mass] (or given charge or angular momentum). Which in turn is closely related to the isoperimetric problem of minimizing area for given volume.

As a measure of size in the left-hand side of (201), one may attempt, inspired by the quasilocal inequalities for black holes (Sect. 4), to use the area of the surface enclosing the object. However, there are counter examples to an inequality of the form $A\ge 4\pi Q^2$. The electrically counterpoised spheroids of dust, presented by Bonnor (1998), are regular, static, isolated systems that satisfy the energy conditions and whose enclosing surface can be made arbitrarily small relative to the charge enclosed, namely, $A<kQ^2$ for any positive, arbitrary number *k*. Since these objects are highly prolate, it is expected that by assuming some kind of roundness on the enclosing surface, the area may give the desired estimate of charge. This example does not mean that the area should not be considered as a measure of size for the not round enough objects. But it says that in some cases, the area alone is not enough to control the amount of charge the object can carry.

Taking this observation into account, two paths can be taken to arrive at estimates of the form (201). One is to use special surfaces that are round enough. The other possibility is to use a measure that takes into account the deformation away from sphericity. In the first approach we encounter the following surfaces that capture the notion of round enough surface: isoperimetric surfaces (Sect. 5.2.3) coordinate spheres and convex surfaces (Sect. 5.2). Within the second approach, one may, as a first step, seek estimates using combinations of different well known measures, like area, distance to the boundary, etc. We come back to this point in Sect. 5.2.

There is another important issue referred to ordinary objects that is closely related to black holes, and it is the question of the collapse of an object to form a black hole. A few black hole formation criteria were constructed from geometrical inequalities stating that if certain inequality is not satisfied, then a black hole is formed. We review them below.

### Results

As is the case for black holes, we divide the results according to whether angular momentum is considered explicitly into the inequality or not. This has to do mainly with the requirement of axial symmetry needed to define quasilocal angular momentum. As we see below, some of the results for ordinary objects employ similar techniques as the ones used in the treatment of black holes, i.e., harmonic maps theory, inverse mean curature flow, stability conditions, etc. Some results are quasilocal and some are global as they also incorporate the ADM mass. Various approaches have been taken to obtain the estimates. However, no variational problem has been formulated. This, in particular, implies that there is no (non-trivial) rigidity statement on the inequalities.

We mention here the setting where these inequalities are proven, and the main properties that lead to them.

*Settings.* All inequalities presented in this section are proven for objects in an initial data set $(\varSigma , h_{ij}, K_{ij}, \mu ,j^i)$. The objects themselves are taken to be open, bounded regions $\varOmega \subset \varSigma$ with smooth boundaries $\partial \varOmega$.

*Inequality producer*. The various results we show in Sects. 5.2.1 and 5.2.2 use very different and apparently unrelated conditions that translate into the found inequalities.

- Strict positivity of the first eigenvalue of the linear differential operator $-\varDelta +\frac{1}{2}{}^3R$.
- Stability of minimal surfaces and MOTSs.
- Stability of the quotient space of maximal slices in axial symmetry.
- Positivity and monotonicity properties of the Geroch energy.
- Stability of isoperimetric surface.

#### Inequalities for objects without angular momentum

 Schoen and Yau (1983) study the black hole formation problem. They consider a maximal initial data $(\varSigma , h_{ij}, K_{ij}, \mu , j^i)$ and an open subset $\varOmega \subset \varSigma$ (the object) such that $\mu \ge \lambda >0$ on $\varOmega$, where $\lambda$ is a constant. Then

202 $$\begin{aligned} {\mathcal {R}}_{SY}^2(\varOmega )\le \frac{\pi }{6\lambda } , \end{aligned}$$

where the radius ${\mathcal {R}}_{SY}(\varOmega )$ is defined as follows. Take a simple closed curve $\varGamma$ in $\varOmega$ which bounds a disk in $\varOmega$. Let *r* be the greatest distance from $\varGamma$ such that the set of all points within this distance form a torus embedded in $\varOmega$. ${\mathcal {R}}_{SY}(\varOmega )$ is the supremum of this *r* over all curves $\varGamma$.

The key point in this result is the fact that the first Dirichlet eigenvalue of the operator $-\varDelta +\frac{1}{2}{}^3R$, if it is strictly positive, sets an upper bound to ${\mathcal {R}}_{SY}$.

Inequality (202) is purely quasilocal, the initial data does not need to be asymptotically flat.

Shoen and Yau also obtain the following black hole formation criterion: If $\varSigma$ is asymptotically flat and matter fields satisfy the energy condition $\mu -|j|\ge \lambda >0$ on $\varOmega \subset \varSigma$, then the opposite inequality to (202) implies that $\varSigma$ contains an apparent horizon.

As stated by Murchadha (1986), the radius ${\mathcal {R}}_{SY}$ captures the idea that the object must be large in every direction to avoid collapsing, but may be hard to compute in practical situations. He defines a new size measure (Murchadha 1986) as follows. ${\mathcal {R}}_{OM}$ is the size of the largest stable minimal 2-surface $\mathcal {S}$ that can be embedded in $\varOmega$. By size we mean the maximum of the distances (with respect to $h_{ij}$) from interior points to the boundary $\mathcal {S}$. The existence of such minimal surfaces is guaranteed when $\varOmega$ is mean convex (i.e., $\partial \varOmega$ has positive mean curvature). He finds ${\mathcal {R}}_{OM}\ge {\mathcal {R}}_{SY}$ and obtains a sharpened version of (202), that is

203 $$\begin{aligned} {\mathcal {R}}_{OM}^2(\varOmega )\le \frac{\pi }{6\lambda } . \end{aligned}$$

Galloway and O’Murchadha (2008) generalize the above result to not necessarily maximal initial data and with MOTS replacing the minimal surfaces. More precisely, they consider an object to be a relatively compact null mean convex open set $\varOmega$ with connected boundary in an initial data set $(\varSigma , h_{ij}, K_{ij}, \mu , j^i)$. Define the radius of $\varOmega$, ${\mathcal {R}}_{GOM}(\varOmega )$ as the size of the greatest compact connected stable MOTS $\mathcal {S}$ contained in $\varOmega$ (size has the same meaning as in the O’Murchadha’s definition of ${\mathcal {R}}_{OM}$). Then, assuming $\mu -|j|\ge \lambda >0$ with $\lambda$ constant, obtain

204 $$\begin{aligned} {\mathcal {R}}_{GOM}^2(\varOmega )\le \frac{\pi }{6\lambda } . \end{aligned}$$

Note that the convexity condition is needed to guarantee the existence of the MOTSs in $\varOmega$ (Eichmair 2007).

We wish to remark that inequalities (203) and (204) use stable minimal or trapped surfaces inside the object under study. In this way, they introduce the positivity condition which ultimately produces the inequality.

Reiris (2014b) shows that the quotient space of maximal slices in axial symmetry satisfies a stability property. This is an interesting and strong argument which gives the desired positivity condition similar to that of stable minimal surfaces on the ambient space. The well known techniques of minimal surfaces are then adapted to obtain a similar bound to that of Schoen and Yau in spherical symmetry. Namely, in spherically symmetric and asymptotically flat initial data

205 $$\begin{aligned} {\mathcal {R}}_A^2(\varOmega )\le \frac{2\pi }{3\lambda } , \end{aligned}$$

where $\lambda$ is a positive constant bounding the energy density, $\lambda \le \mu$, and ${\mathcal {R}}_A(\varOmega )$ is the areal radius of the constant radius sphere $\varOmega$.

This result gives also the following black hole existence criterion: If the energy density of the object satisfies $\rho >\pi /6M^2$ where *M* is the ADM mass, then the object lies inside a black hole and is not in static equilibrium.

He also obtains

206 $$\begin{aligned} {\mathcal {R}}_A\ge \frac{Q^2}{2M_{ADM}} \end{aligned}$$

for spherically symmetric, asymptotically flat initial data, satisfying the dominant energy condition.

Khuri (2015b) decomposes the matter density as an electromagnetic part (subindex *EM*) and a non-electromagnetic part (subindex *M*) as $\rho =\rho _{M}+\rho _{{\textit{EM}}}$ and $j=j_{M}+j_{{\textit{EM}}}$ and assumes that the non-electromagnetic part satisfies the dominant energy condition, that is $\mu _M\ge |j_M|$. Moreover, $\mu _M$ is taken to be constant. Then from this condition and the definition of electromagnetic charge he obtains

207 $$\begin{aligned} Q^2\le \frac{A}{2\pi }\int _{\partial \varOmega }\mu _M-|j_M| , \end{aligned}$$

where *A* is the area of $\partial \varOmega$. Then, using the Schoen and Yau bound (202) he obtains

208 $$\begin{aligned} |Q|\le \frac{A}{\sqrt{12}{\mathcal {R}}_{SY}} \end{aligned}$$

Moreover, if $\varOmega$ is mean convex, then the same bound holds for ${\mathcal {R}}_{OM}$ instead of ${\mathcal {R}}_{SY}$.

Anglada et al. (2016) study the spherically symmetric, electrovacuum case. They find that if the initial data is asymptotically flat and spherically symmetric, and if outside a ball $\varOmega$ with finite areal radius ${\mathcal {R}}_A$, it is electrovacuum and untrapped, then

209 $$\begin{aligned} {\mathcal {R}}_A\ge \frac{|Q|}{2} . \end{aligned}$$

This inequality is weaker than (206) and it is saturated at $Q=0$ with vanishing radius and total mass. Moreover, every non-trivial objet satisfies the strict inequality. (209) is not quasilocal in the sense that asymptotic flatness is required for (209) to hold and, in fact there are non-asymptotically-flat examples were it is violated. Note that (209) does not use the bound (202) nor the radius ${\mathcal {R}}_{SY}$, as ${\mathcal {R}}_A$ is a more natural size measure in spherical symmetry (see the discussion about the convenience of using the surface area as a size measure for objects, in Sect. 5.1).

#### Inequalities for objects with angular momentum

The key ingredient needed to include the angular momentum into a geometrical inequality is to relate it with the Einstein constraints. This is done via the relation with the current density $j^i$ or with the extrinsic curvature $K_{ij}$ of $\varSigma$.

Dain (2014b) considers maximal axially symmetric initial data $(\varSigma , h_{ij}, K_{ij}, \mu , j^i)$ with constant energy density $\mu$ and non-vanishing current density $j^i\ne 0$, satisfying the dominant energy condition. He takes an object to be an axially symmetric open subset $\varOmega$ of $\varSigma$. From the definition of angular momentum [see Eq. (138)] he bounds the angular momentum *J* of $\varOmega$ in terms of the integral of the current density (and via the energy condition, in terms of $\mu$) and the norm of the Killing vector $\eta ^i$ associated to the axial symmetry, that is $\eta =\eta ^i\eta _i$,

210 $$\begin{aligned} |J|\le \int _\varOmega |j|\sqrt{\eta }\le \mu \int _\varOmega \sqrt{\eta }. \end{aligned}$$

Then, using the Hamiltonian constraint he obtains $R\ge 16\pi \mu$, and therefore, (202) gives the geometrical inequality between the angular momentum *J* of $\varOmega$ and the Schoen and Yau size.

211 $$\begin{aligned} |J|\le \frac{\pi }{6}\frac{\int _\varOmega \sqrt{\eta }}{{\mathcal {R}}_{SY}^2}. \end{aligned}$$

In fact, Dain proposes the right-hand side of (211) as a new measure of size.

By assuming $\varOmega$ to be mean convex, the same inequality is obtained when ${\mathcal {R}}_{OM}$ is used in the definition of ${\mathcal {R}}_D$.

Khuri (2015a) extends this result to not necessarily maximal initial data satisfying a stronger version of the dominant energy condition, namely $\rho \ge |{\bar{j}}| +|j_\eta |$ where $j_\eta$ is the current density in the direction of the axial Killing vector field $\eta ^i$ and ${\bar{j}}$ is the current in the orthogonal directions. More precisely, Khuri considers an axially symmetric initial data, without compact apparent horizons, which is asymptotically flat or has a strongly untrapped boundary, then for an open set $\varOmega$ in the initial data it holds

212 $$\begin{aligned} |J|\le \frac{3\pi C_0}{16}\frac{\int _\varOmega \sqrt{\eta }}{{\mathcal {R}}_{SY}^2}. \end{aligned}$$

where $$C_0:=\max _\varOmega (\mu -|{\bar{j}}|)/\min _\varOmega (\mu -|{\bar{j}}|)$$.

This result gives the following black hole formation criteria: Given an axially symmetric initial data such that the initial surface $\varSigma$ is asymptotically flat or has a strongly untrapped boundary. Under the energy condition stated above, if there exists a bounded region $\varOmega$ where (212) does not hold, then $\varSigma$ contains an apparent horizon.

Without using the Schoen and Yau bound (202), Reiris (2014b) goes a different route to obtain geometrical inequalities for objects. Using techniques of minimal surfaces he finds estimates on the shape of an axially symmetric object in terms of the angular momentum when the object does not intersect the symmetry axis and is connected:

213 $$\begin{aligned} |J|\le \left( 1+\frac{P}{\pi D}\right) \frac{\pi }{2}{\mathcal {R}}_C^2 , \end{aligned}$$

where *P* is the transversal perimeter of $\varOmega$, *D* is the distance from $\varOmega$ to the symmetry axis and $2\pi {\mathcal {R}}_C$ is the length of the greatest axisymmetric orbit in $\varOmega$. Note that this result gives information, not only about the size, but about the shape of the object. This implies that in order to control the angular momentum of an ordinary object, size in all directions should be considered, an observation that may also be valid for electrically charged ordinary objects (see the discussion about the Bonnor example mentioned in Sect. 5.1).

The inequalities (211), (212) and (213) suggest the existence of an appropriate size measure, ${\mathcal {R}}$, probably defined in terms of the norm of the Killing vector $\eta$, as well as measures in relevant spatial directions, such that an inequality of the form

214 $$\begin{aligned} |J|\lesssim {\mathcal {R}}^2 \end{aligned}$$

holds for ordinary objects.

We finally present an inequality that is global in the sense that in includes the ADM mass.

Using the inverse mean curvature flow on asymptotically flat, axially symmetric initial data $(\varSigma , h_{ij}, K_{ij}, \mu , j^i)$, Anglada et al. (2017) study convex regions $\varOmega$ where the current density has compact support. Assuming that the initial data satisfies the dominant energy condition and has no minimal surfaces, they find

215 $$\begin{aligned} M_{ADM}\ge m_T+\frac{J^2}{5{\mathcal {R}}_A{\mathcal {R}}_c^2} \end{aligned}$$

where ${\mathcal {R}}_A$ and ${\mathcal {R}}_c$ are the areal and circumferential radius of the convex flow surface $S_T$ such that $S_t$ is convex for $t\ge T$. Also, $m_T$ is a positive constant

216 $$\begin{aligned} m_T:=\frac{1}{16\pi }\int _0^{{\mathcal {R}}_A}d\xi \int _{S_\xi } RdS \end{aligned}$$

and $\xi$ is the areal radius coordinate and *R* is the curvature scalar of *h*.

One of the main ingredients in the argument is the use of the Geroch energy (Huisken 1998; Szabados 2004) defined on a 2-surface *S* with area *A* and mean curvature *H*

217 $$\begin{aligned} E_G(S):=\frac{A^{1/2}}{(16\pi )^{3/2}}\left( 16\pi -\int _{S}H^2ds\right) . \end{aligned}$$

The positivity and monotonicity properties of the Geroch energy are crucially used to relate the ADM with the curvature scalar and the norm of the Killing vector field, $\eta$, on the surfaces defined by the flow. Using the Hamiltonian constraint together with the definition (139), the scalar curvature is bounded by the angular momentum of the surfaces. Finally convexity of the flow surfaces is used to control the evolution of $\eta$ along the flow.

Inequality (215) also gives information about the shape of the object. It says that if the total mass is fixed, then the angular momentum determines how oblate or prolate the object can be. We also notice that the term $m_T$ plays the role of a quasilocal mass (see Malec et al. 2002).

It is remarkable that the inequalities obtained in this section, although with different technical conditions, give rise to inequalities similar to the ones discussed in Sect. 1.3, which were informally derived from Newtonian considerations and the condition that nothing travels faster than light.

#### Isoperimetric surfaces

As we mention in Sect. 2.2, isoperimetric surfaces in initial data are an important system from which relations between physical and geometrical quantities can be obtained. We refer the reader to the articles by Eichmair and Metzger (2013a, b) for a detailed account on the results related to isoperimetric surfaces in Riemannian manifolds with application to General Relativity. See also Sect. 2.2 for references on discussions about the Penrose inequality for isoperimetric surfaces.

In this section we focus on inequalities relating size, angular momentum and charges, so, in this sense, they are quasilocal inequalities.

Dain et al. (2012) study electro-vacuum, maximal initial data, possibly with a non-negative cosmological constant and find that if $\mathcal {S}$ is a stable isoperimetric sphere, then

218 $$\begin{aligned} A(\mathcal {S})\ge \frac{4\pi }{3} Q^{2}(\mathcal {S}) . \end{aligned}$$

Stability here means that the area function is not only critical at the isoperimetric surface $\mathcal {S}$, but also a minimum.

Aceña and Dain (2013), characterize the behavior of isoperimetric surfaces in Reissner–Nordström and find, among other results that the spheres $r=constant$ in the Reissner–Nordström metric are isoperimetric stable for $0\le |Q|\le M$ and satisfy the bound

219 $$\begin{aligned} A \ge \frac{16}{9}\pi Q^2 . \end{aligned}$$

Moreover, there is not a sphere in Reissner–Nordström where the inequality (219) is saturated. The inequality is saturated in the limit when the extreme case is approached from the superextreme case.

Up to now, the only result involving angular momentum for isoperimetric surfaces is proven by Reiris (2014b). Let $\mathcal {S}$ be a stable isoperimetric, axisymmetric sphere enclosing an object $\varOmega$ (and nothing else). Then,

220 $$\begin{aligned} |J|\le c_1 R \sqrt{A} \le c_22 R L , \end{aligned}$$

where $c_1 = 6/(8\pi 3/2)$, $c_2 = 6/(4\pi )$, |*J*| is the angular momentum of $\varOmega$ and *A*, *R* and *L* are, respectively, the area of $\mathcal {S}$, the length of the greatest axisymmetric orbit in $\mathcal {S}$ and the distance from the North to the South Pole of $\mathcal {S}$.

## Open problems

There are a number of open problems that need to be addressed in order to understand more completely the type of estimates one can obtain both for black holes and ordinary objects. Most of them were mentioned and/or discussed in the appropriate section. We list them below as well.

- *Removing axial symmetry* Axial symmetry is a requirement in all the geometrical inequalities involving angular momentum presented in this article. However, deviations from axial symmetry are of major importance, especially in astrophysics and numerical simulations. As was previously discussed, axial symmetry is an unremovable condition for the global inequalities (Huang et al. 2011). However, for quasilocal inequalities, the necessity of axial symmetry is not so clear. On one hand, there are heuristic and Newtonian-like considerations that suggest the possibility of non-axially symmetric quasilocal geometrical inequalities. Also, the fact that the variational problem for the mass functional $\mathbb {M}$ presented in Sect. 4.2.5 holds for not necessarily axially symmetric functions is somewhat encouraging. In particular, this last point shows the major role that extreme Kerr–Newman black holes plays as a limit solution, among a wider class of solutions. We need to emphasize that the problem of quasilocal geometrical inequalities involving angular momentum outside axial symmetry is not a well defined mathematical problem, because there is no proper quasilocal notion of angular momentum in this setting. Solving this problem, for one way or another is a major open problem.
- *Maximality and Jang-like equations* As we have seen in several parts of this article, most inequalities for initial data were originally proved assuming maximality. Whether this condition could be lifted or not was an unsolved question for some time. Later maximality was replaced by the existence of solutions to especific equations including equations of the Jang type. To prove that such solutions do exist is key to regard the maximality condition a removable one.
- *Mass–angular momentum inequality for data with inner boundary* We have seen in Sect. 3.1 that the mass–charge inequality can be formulated in terms of initial data where the initial surface $\varSigma$ is either complete with non-trivial topology, or has a weakly trapped inner boundary. On the other hand, the mass–angular momentum inequalities presented in Sect. 3.2 are proven only for complete initial surfaces with non-trivial topology. Extending this result to manifolds with boundaries is important for three reasons: first it would complete and unify the results about this type of global inequalities. Second, the proper formulation and resolution of the variational problem needed to derive the desired inequality when an inner boundary is present (analogous to the one used in the proof for the case of complete manifold), would clarify the role that extreme black holes play as borderline solutions. Finally, it seems that if one wants to make a connection between this geometrical inequality and the Penrose inequality, (see next item) a careful understanding of this case may be of use.
- *Connection with Penrose inequality* The connection of the geometrical inequalities presented in this article and the positive mass theorem and the Penrose inequality seems to become deeper as further studies are performed. Not only they involve the same physical and geometrical quantities, i.e., mass, area (or size), angular momentum, charge, but also the techniques used in both problems seem to not be so different (see Sect. 5.2 for an inequality for objects using the inverse mean curvature flow). Exploring this connection may shed light into the problems and possible resolutions. In this respect, see the article by Anglada (2017), where he adapts the results in Anglada et al. (2017) for ordinary objects to study the Penrose inequality.
- *Minimum of mass functional for multiple rotating black holes* The global inequality for multiple black holes, (147) is written in terms of the value of the mass functional on a minimizer solution. This minimizer is not known explicitly although there are a few numerical calculations indicating that for two regular Kerr black holes, the total mass square should be greater than the total angular momentum. Whether this relation holds for more general configurations or some other parameters are needed is unclear. Obtaining an explicit form of this value is of great importance because it would tell us, in particular, whether the total mass controls the individual angular momenta of the black holes, or the total angular momentum of the system, and exactly how it does it.
- *Global inequality with *$\varLambda$ Global inequalities relating mass, angular momentum and/or charge that also include explicitly a cosmological constant $\varLambda$ have not been proven yet. A negative cosmological constant, however, has been admitted in the statements of the mass–angular momentum–charge inequalities (Cha and Khuri 2017).
- *Connection between *$\mathcal {M}$ *and* $\mathbb {M}$ Another issue that must be better understood is the connection between the global and quasilocal inequalities for black holes that include the angular momentum. A partial implication was presented in Sect. 4.3 but there are many issues that are not entirely clear yet. This is not an easy problem since it involves relating global and quasilocal settings. Its full resolution might give a hint into the connection with the Penrose inequality.
- *Quasilocal estimate with negative *$\varLambda$ The way the cosmological constant appear into the mass functional $\mathbb {M}$ makes the procedure used to prove the area–angular momentum–cosmological constant inequality hard to adapt when $\varLambda$ is negative. Note that the problem with the negative $\varLambda$ is not about how it enters into a generalization of the Area–Angular momentum inequality, but about whether such an inequality does exist. The works mentioned in Sect. 4.4 suggest that it does exist and a particular inequality motivated by extreme Kerr–Newman AdS black hole has been proposed. This problem is far from solved and new techniques must be implemented.
- *Ordinary objects* As was discussed in Sect. 5, there are very basic questions that are unanswered with respect to geometrical inequalities for objects. Things like: what inequality we expect to obtain, how we should characterize the object and how we should measure them, are not clear. Concerning the first two issues, it is crucial to understand in what class of ordinary objects one expects to obtain a geometrical inequality. By this we mean that a positivity condition seems to be needed in order to derive the desired estimate. This leads naturally to the following question: Do all objects, say, in axial symmetry for simplicity, have a restriction on the allowed valued of their parameters? In particular, should they be *round enough*?
- *Measure of size for ordinary objects* This issue was discussed in Sect. 5. There are various alternative notions of size but more work needs to be done. As seen in the results presented in Sect. 5.2.2, in the case of axial symmetry, it may be convenient to study measures constructed from the norm of the axial Killing vector field, $\sqrt{\eta }$. This is supported by the following observations: The norm $\sqrt{\eta }$ is bounded by the equatorial radius ${\mathcal {R}}_c$ (defined as the length, divided by $2\pi$ of the greatest axially symmetric circle. This gives a clear and natural measure of size relevant for rotating objects. Also, for convex surfaces the variation of $\eta$ along the inverse mean curvature flow is controlled by $\eta$ itself. Also, the measure should take into account deviations from sphericity in all directions.
- *Connection with hoop conjecture* Some versions of the hoop conjecture suggest to look for geometrical inequalities relating size, angular momentum and some measure of quasilocal mass of a certain region of spacetime (Senovilla 2008). There are several different quasilocal masses in the literature (Szabados 2004), but the problem of identifying the appropriate one(s) that simultaneously capture the matter content of the region, and that give rise to the desired meaningful geometrical inequalities is still open.

## Footnotes

1 We thanks L. Szabados for clarifying this point.
